# Advances in Physiologically Based Pharmacokinetic (PBPK) Modeling of Nanomaterials

**DOI:** 10.1021/acsptsci.4c00250

**Published:** 2024-07-12

**Authors:** Ozlem Ozbek, Destina Ekingen Genc, Kutlu O. Ulgen

**Affiliations:** Chemical Engineering Department, Bogazici University, Bebek 34342 Istanbul, Turkey

**Keywords:** physiologically based pharmacokinetic (PBPK) model, nanoparticles (NPs), absorption, distribution, metabolism, excretion (ADME), interspecies scaling, *in vitro* to *in vivo* extrapolation (IVIVE), artificial intelligence (AI)

## Abstract

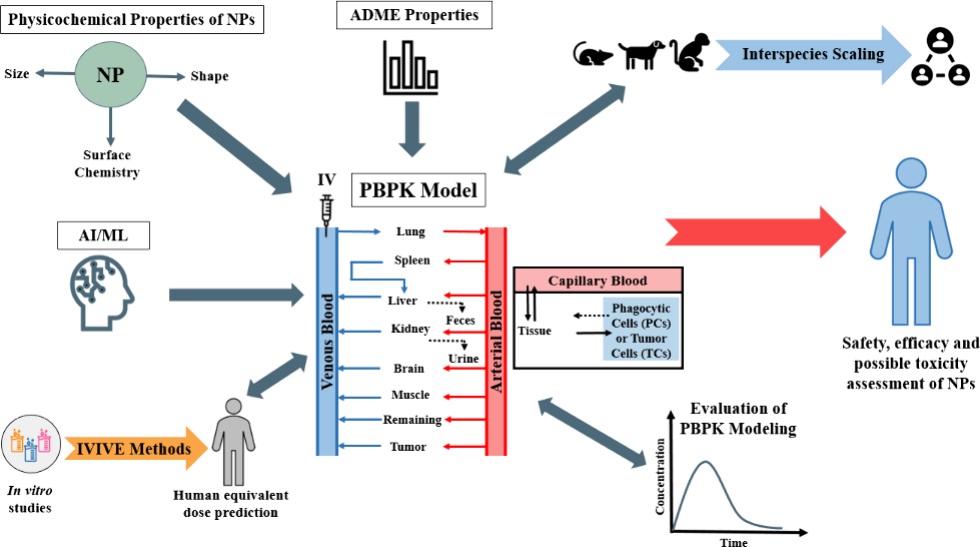

Nanoparticles (NPs) have been widely used to improve the pharmacokinetic properties and tissue distribution of small molecules such as targeting to a specific tissue of interest, enhancing their systemic circulation, and enlarging their therapeutic properties. NPs have unique and complicated *in vivo* disposition properties compared to small molecule drugs due to their complex multifunctionality. Physiologically based pharmacokinetic (PBPK) modeling has been a powerful tool in the simulation of the absorption, distribution, metabolism, and elimination (ADME) characteristics of the materials, and it can be used in the characterization and prediction of the systemic disposition, toxicity, efficacy, and target exposure of various types of nanoparticles. In this review, recent advances in PBPK model applications related to the nanoparticles with unique properties, and dispositional features in the biological systems, ADME characteristics, the description of transport processes of nanoparticles in the PBPK model, and the challenges in PBPK model development of nanoparticles are delineated and juxtaposed with those encountered in small molecule models. Nanoparticle related, non-nanoparticle-related, and interspecies-scaling methods applied in PBPK modeling are reviewed. *In vitro* to *in vivo* extrapolation (IVIVE) methods being a promising computational tool to provide *in vivo* predictions from the results of *in vitro* and *in silico* studies are discussed. Finally, as a recent advancement ML/AI-based approaches and challenges in PBPK modeling in the estimation of ADME parameters and pharmacokinetic (PK) analysis results are introduced.

With the expanding interest in the discovery and development of drug delivery systems containing nanoparticles, the need for effective quantitative tools to determine the safety, efficacy, and possible toxicity of these materials has emerged. Physiologically based pharmacokinetic (PBPK) modeling is a computational tool to simulate the absorption, distribution, metabolism, and elimination (ADME) characteristics of the materials that are administered inside the body of living organisms. PBPK models have the ability to simulate the drug concentration profile in the targeted site of action such as specific organs or tissues that facilitates the adjustment of a dosage strategy and helps to attain maximum safety and efficacy profiles.^1^ PBPK models are comprised of a series of differential mass balance equations which stand for the physiological processes that occur in the biological tissues or fluids of an organism.

PBPK modeling serves as a valuable tool for forecasting and unraveling the dynamics of drug behavior by both mechanistically figuring out the disposition of the drug and empirically by parameter estimation.^2^ The major requirements in the formation of a PBPK model are the species-specific anatomical and physiological data and also the compound-specific pharmacokinetic data and partition coefficients in different tissues.^3^ Once the substance-specific and species-specific parameters are obtained, PBPK models can be developed for organic and inorganic compounds. The building blocks or the compartments in the construction of PBPK models are tissues in the body such as the heart, liver, kidney, brain, gut, spleen, lung, muscle, and adipose tissues. Unless they are not related to the organ of interest, the other tissues in the body may be grouped in a remainder compartment, or they may be neglected if they are not important concerning mass balance. For simplification, tissues that have comparable kinetic properties can be lumped together.^4^ In PBPK models, all the tissue compartments are linked to the circulating blood system and occasionally by the lymphatic system. The drug-metabolizing tissue compartments such as the liver and kidney are selected to define drug clearance. Species extrapolation, dose–response prediction, risk assessment, and individual variability prediction can be mentioned among the advantages of PBPK models. Integrating genetic and physiological variations with physicochemical properties, PBPK models can predict individual differences in response to toxicants, aiding personalized risk assessments and interventions.

The discovery and the development of nanoparticle-based drug delivery systems have been a research subject of major interest owing to their various advantages in the field of targeted drug delivery. Nanoparticle-based drug delivery systems have been considered promising agents in the treatment of various pathologies such as cancer, diabetes, neurodegenerative, cardiovascular, and respiratory and infectious diseases^5−7^ and also have been widely used as diagnostic and imaging agents for the early diagnosis of disease.^5,8−10^ Nanoparticles which have various sizes, surface charges, and functionalization can exhibit different pharmacokinetic behaviors that may limit the preclinical assessments when compared with conventional therapeutics.^11^ In this aspect, the use of computational and mathematical modeling that are discrete such as quantum mechanics, molecular dynamics, or continuous models like physiologically based pharmacokinetic (PBPK) modeling and pharmacokinetic/pharmacodynamic (PK/PD) modeling stands as promising tools to predict the pharmacokinetic properties of nanoparticles.

In this review, recent advances in PBPK model applications focusing on nanoparticles and considering their unique characteristics, their dispositional features in the biological systems, ADME characteristics, the description of transport processes of nanoparticles in the PBPK model, and the challenges in PBPK model development of nanoparticles are summarized and compared with those of the small molecules. PBPK modeling and simulation have been widely used to predict the pharmacokinetics (PK) and disposition of small molecules and biologics; however, these practices to nanoparticles are bounded and challenging because of the complicated *in vivo* transport mechanisms, for instance, opsonization and uptake by mononuclear phagocyte system (MPS), recognition and internalization by the cells, enhanced permeability and retention (EPR) effect, lymphatic transfer, enzymatic degradation and structural property changes in biological systems.^12^

This review provides an overview of the ADME characteristics of nanoparticles and parameters used in PBPK modeling including interspecies scaling techniques, and gives brief summary on the recent developments regarding the PBPK modeling of nanoparticles. The use of *in vitro* to *in vivo* extrapolation (IVIVE) techniques integrated with PBPK models to determine the safety, efficacy, and potential toxicity of a rapidly increasing number of nanoparticle-based products is presented. These techniques are considered as promising approaches in the risk estimation of nanoparticles owing to being nonanimal alternative techniques to the traditional methods. The major challenges in the model development and validation of nanoparticle based PBPK models are also discussed. Finally, as a novel technique in PBPK modeling, AI (Artificial Intelligence)-Assisted PBPK modeling and related literature studies are introduced. With the considerable progression in the field of nanotechnology and nanomedicine over the past few decades, efficient development of modeling frameworks using novel applications that facilitate the prediction of species-specific physiological disposition has been emerging to extrapolate both from *in vitro* to *in vivo* as well as from animals to humans. This paper outlines the advancements and challenges and points out the future prospects in the PBPK modeling of nanomaterials.

## ADME Characteristics of Nanoparticles

**Figure 1:**
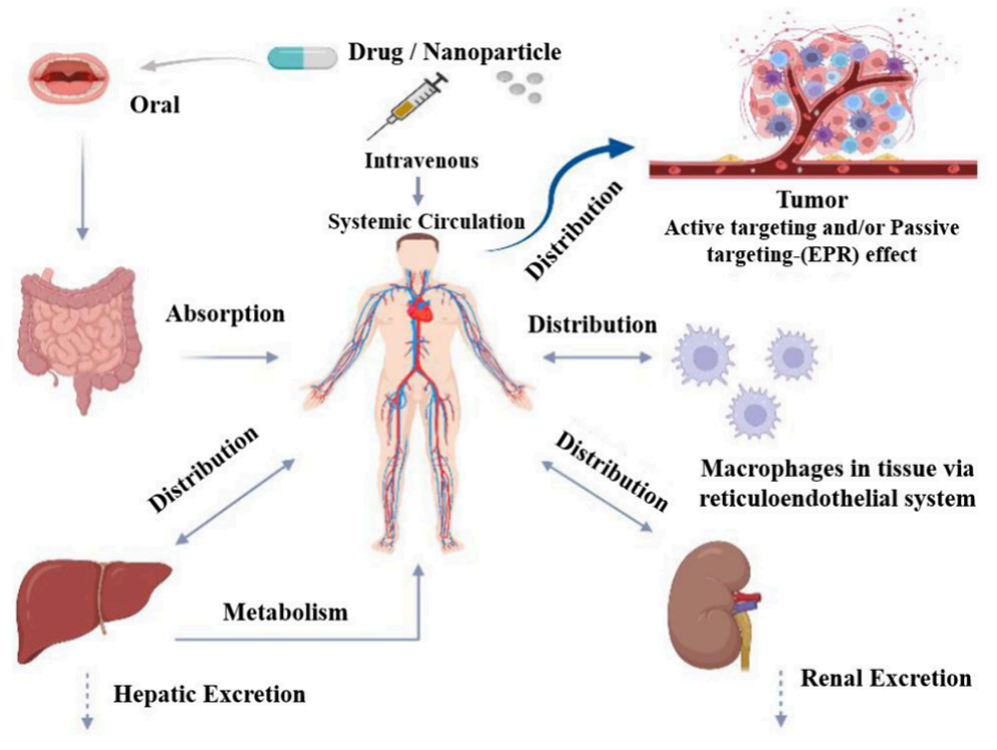
Adsorption, distribution, metabolism, and excretion (ADME) of drugs and/or nanoparticles. Reproduced with permission from ref (26) with no modifications. Open Access. CC-BY 4.0 license. Copyright: © 2022 by the authors. Licensee MDPI, Basel, Switzerland. URL: https://www.mdpi.com/2075-4426/12/5/673. No endorsement.

Unlike the PBPK modeling of small molecules, the unique interaction of the nanoparticles with physiological systems should be considered when constructing PBPK models for nanoparticles.^12^ The limitations in the development and the validation of the PBPK models of NPs are regarded as a deficiency in the reliable and rapid analytical approaches for NP models.^13^ This situation arises from the much more complex ADME properties of NPs compared to the small molecules and also the difficulty in the NP isolation from the tissues compared to isolating from the plasma samples.^13^ In this aspect, it is crucial to incorporate the ADME properties of the nanoparticles specifically according to their features into the PBPK models, to enhance the predictability and performance of these models. A schematic representation of the adsorption, distribution, metabolism, and excretion (ADME) of drugs and nanoparticles is shown in Figure 1.

### Absorption

Most of the nanoparticles designed for drug targeting have been intended to be administered by intravenous (IV) injection, and many of them have been developed to extend the circulation time or target the organ specific to that disease through the circulation system.^4^ When the nanoparticles are administered via nonintravenous injection methods, they go through two competitive processes that are preabsorption clearance and absorption.^4^ In preabsorption clearance, nanoparticles encounter local degradation and direct removal by different excretion routes upon oral, pulmonary, and nasal administration.^4^ In the local degradation of NPs, the release of an active pharmaceutical ingredient (API) from an encapsulated form of NP can take place at the administration site, and APIs are disposed like small molecules.^4^ After oral administration, the nanoparticles that do not encounter preabsorption clearance can participate in the bloodstream and lymphatic system by crossing the unstirred water layer and the epithelium of the gastrointestinal (GI) tract.^4^ After pulmonary exposure, nanoparticles deposited in the lungs can be exhaled, discharged via mucociliary clearance to the GI tract, or kept apart and degraded by macrophages.^4^ The nanoparticles left from these discharge mechanisms can be absorbed after crossing the mucus and lung epithelium cells.^4^ After subcutaneous, intramuscular, intradermal, or intraperitoneal injections, NPs are mostly absorbed via highly permeable lymph vessels or via macrophages and transferred into regional lymph nodes.^4^

### Distribution

Nanoparticles pass to the vascular system after intravenous administration and are then distributed to the tissues and organs of the body.^3^ After the NP administration via inhalation, NPs are placed in the alveolar region and taken up by the alveolar macrophages.^3^ These alveolar macrophages then move to the tracheobronchial region with the aim of mucociliary clearance or to the mediastinal lymph nodes.^3^ Some of the nanoparticles may enter to epithelium via the process of endocytosis.^3^ The region-specific placement information on the nanoparticles obtained from the model can be useful in the determination of the multicompartmental parameters in PBPK modeling.^3^ When the nanoparticles arrive at the vascular system, they come across blood cells, plasma proteins, platelets, and coagulation factors.^3^ The proteins present in the vascular system are adsorbed on the surface of these nanoparticles, and as a result, NP-protein complex and dynamic corona formation occur.^3^ The NP-protein corona affects the internalization and the distribution of the nanoparticles into different tissues.^3^ It is imperative to incorporate the formation of NP-protein complexes and their dynamic processes when constructing a PBPK model.^3^ Since the mechanism inside the NP-protein formation process has not been fully revealed,^14^ it is significant to carry out further studies on this topic.

### Metabolism

The enzymatically degradable nanoparticles (such as protein, lipid, and poly(lactic-*co*-glycolic) acid (PLGA) based NPs) can be metabolized inside the system of the organism, whereas inorganic nanoparticles (nondegradable in general) are not metabolized.^3^ On the other hand, the ligands that are used as a surface modification of these inorganic nanoparticles and organic coatings can be degraded enzymatically.^3^ Most of the xenobiotics are metabolized in the liver (the main organ responsible for the metabolism), and the enzymes that participate in the metabolism belong to the transferase, esterase, monooxygenase and epoxide hydrolase families.^3^ However, the nanoparticles which are hard to degrade by intracellular processes like inert Au NPs remain inside the cells and are located in the liver for a very long time.^15^ Au nanoparticles, which were administered intravenously, were reported to accumulate in the liver of the mouse, and the duration of the elimination of the nanoparticles lasted more than 6 months.^16^ Also, according to toxicological studies, if the dose of the nanoparticles is higher than the hepatic biodegradation capacity, the residual nanoparticles accumulate in the organ for a long period.^15^ The *in vivo* clearance values of most of the compounds are found by scaling up the *in vitro* metabolic clearance data obtained from human liver microsomes or hepatocytes.^3^ In PBPK modeling, the equations related to the metabolism of compounds are usually described by a combination of linear and Michaelis–Menten kinetic equations.^3^ The kinetic constants of the Michaelis–Menten equation, which are *K*_m_ and *V*_max_ are obtained by *in vitro* or *in vivo* empirical experiments.^3^

Some of the previous studies discarded the pharmacokinetics regarding the metabolism of the nanoparticles that have protein ligands on their surface or include organic coatings since the process is not fully understood.^17^ Hence, in PBPK modeling of NPs, the metabolism term is sometimes described by the processes of dissolution, corona formation, and aggregation/agglomeration.^3^ Since the mathematical description of the dissolution kinetics of nanomaterials has not been fully identified, this process is included in some of the PBPK models and neglected in others. For example, in a study on the PBPK modeling Ag nanoparticles, the metabolic pathway of NPs was first described by the extracellular and intracellular dissolution followed by the release of the soluble silver species and then by the storage of Ag nanoparticles directly as in the form of silver sulfide particles.^18^ In some other studies on the nanoparticles that have low dissolution rates inside the body, e.g., AuNPs, the metabolism process was neglected during the construction of PBPK models.^17^ Moreover, the dissolution of Ag nanoparticles was excluded in the PBPK model of another study, and the results indicated an accurate simulation of nanoparticles.^19^

When the nanoparticles form aggregates or agglomerates, which are regarded as secondary entities, they show more complicated physicochemical properties compared to the single nanoparticles since their properties such as size, surface area, morphology, and effective density change and depend on additional factors.^20^ However, the information on the interaction of the aggregates or agglomerates of the nanoparticles in the biological system has been limited. In a previous article that reviewed the interaction of nanoparticle aggregates with biological systems, the critical threshold particle size for the tissue uptake and clearance was reported to be between 200 and 250 nm, and hence particles larger than this threshold may tend to accumulate.^21^ Compared to larger sized Au nanoparticles (20 nm), smaller sized ones (7 nm) that form aggregates with a maximum diameter of 45 nm showed higher translocation and a wider distribution to secondary organs in rats following 15 days of inhalation exposure.^22^ However, further studies are needed to shed a light on the topic of differences between single particles and aggregated particles at the mechanistic level, and the mechanisms of interaction of the aggregates with the biological system should be identified.

### Excretion

The major organs that are responsible for the excretion process of the nanoparticles are the liver and kidney through feces and urine, respectively.^3^ The nanoparticles can also be excreted from the body through lungs, sweat, and breast milk.^3^ In PBPK models, generally, renal and hepatic clearances of the nanoparticles are considered.^23^ The size of the nanoparticles is significant in renal clearance. For example, quantum dot (QD) nanoparticles smaller than 5.5 nm have been effectively and rapidly excreted from the body via urinary clearance,^24^ whereas for Au nanoparticles with a particle diameter larger than 10 nm the renal excretion process has been neglected.^25^ The pore size of the liver capillaries is much larger than that of kidneys, and hence for nanoparticles the degree of urinary excretion is much smaller than of the biliary excretion.^3^ In this aspect, the hepatobiliary excretion system that includes hepatocytes followed by excretion into the bile is considered as the major clearance route for the larger-sized nanoparticles.^3^ Apart from the size of the nanoparticles, their surface functionalization has been reported to play an important role in biliary excretion; however there is limited knowledge about the effect of surface functionalization of nanoparticles on the excretion process.^3^ Further investigations both based on *in vitro* and *in vivo* assays are significant for the development of accurate PBPK models that represent the excretion process of the nanoparticles.

## Parameters Used in PBPK Modeling

**Figure 2:**
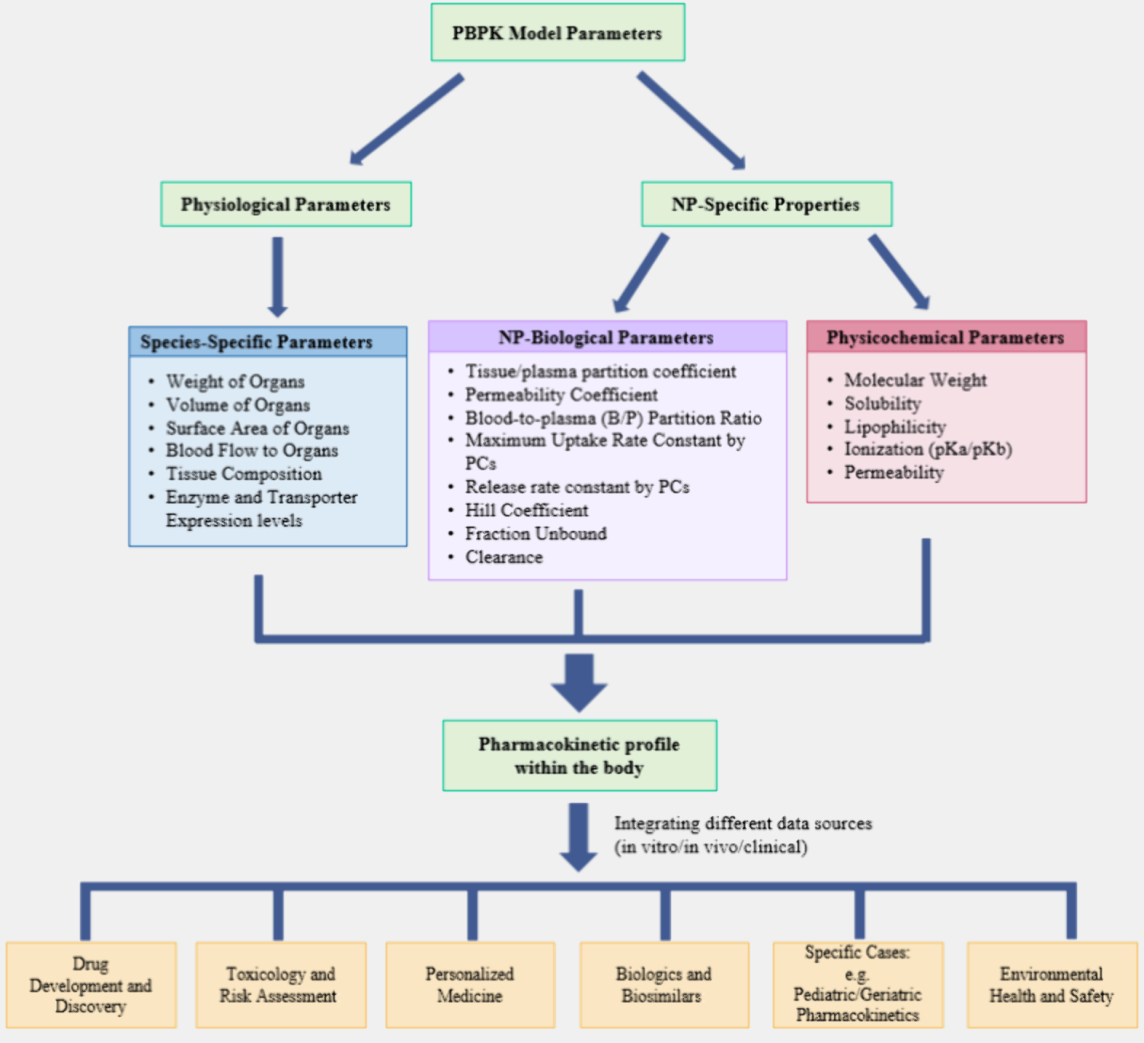
Schematic diagram for PBPK model parameters and application areas.

To predict the absorption, distribution, metabolism, and excretion (ADME) of chemical compounds in living organisms, PBPK models integrate physiological, biochemical, and physicochemical parameters to provide quantitative insights into the kinetic processes of drugs and other substances within the body. The key physiological parameters in PBPK models are the organ volumes (sizes of organs and tissues), the blood flow rates at which blood circulates through various tissues and organs, and the partition coefficients (the ratios of a compound’s concentration between blood and tissue). These parameters along with the enzymatic activities are incorporated into the PBPK models (Figure 2). These parameters are often species-specific, allowing the model to be tailored to different animal species or humans.^27^

The key physicochemical characteristics of toxicants are molecular weight and size influencing the ability of the toxicant to pass through biological membranes and barriers, solubility and lipophilicity showing the compound’s tendency to dissolve in fats, affecting its distribution, ionization (p*K*_a_), and permeability. Water solubility affects how well a toxicant can dissolve in bodily fluids, impacting absorption and excretion, whereas lipid solubility (lipophilicity) determines how readily a toxicant can cross cell membranes and distribute into fatty tissues. The degree of ionization of a toxicant at physiological pH affects its solubility and permeability across cell membranes. Nonionized forms usually permeate cell membranes more readily than ionized forms. The partition coefficients describe the distribution of a toxicant between aqueous and lipid phases, informing its distribution in the body. Higher partition coefficients generally indicate higher lipophilicity, leading to greater accumulation in fatty tissues. The ability of a toxicant to penetrate biological membranes depends on its size, lipophilicity, and degree of ionization.^27^

PBPK models incorporate the physiological aspects of animals as well as the physicochemical properties of toxicants to simulate their absorption through different routes such as oral, inhalation, dermal, or intravenous. For oral absorption, parameters like solubility and permeability through the gastrointestinal (GI) tract are considered. The model simulates the dissolution of the toxicant in the GI fluids and its permeation through the GI lining. For inhalation, factors such as volatility and particle size are integrated to predict the deposition and absorption in the respiratory tract.^27^

The PBPK models predict how the compound distributes across different tissues based on blood flow rates and tissue-specific partition coefficients. This helps in understanding the drug’s or NP’s tissue accumulation and potential target sites. Physicochemical properties are used to determine the distribution of toxicants between blood and tissues. Lipophilic toxicants with high partition coefficients tend to accumulate in fatty tissues, while hydrophilic toxicants remain more in the aqueous compartments. The model accounts for tissue-specific blood flow rates and tissue-to-blood partition coefficients to predict the concentration of toxicants in different tissues over time. PBPK models include metabolic pathways, informed by the physicochemical properties of the toxicant, to predict its biotransformation. The levels and activities of metabolic enzymes help metabolize the compound. Lipophilicity and molecular structure can influence which enzymes metabolize the toxicant and the rate of metabolism. The model can simulate phase I (e.g., oxidation, reduction) and phase II (e.g., conjugation) metabolic processes, taking into account enzyme kinetics and tissue-specific enzyme expression.^27^

The excretion of toxicants is influenced by their solubility and molecular size. Hydrophilic toxicants are typically excreted via the kidneys, while lipophilic ones may undergo biliary excretion. PBPK models simulate renal filtration, reabsorption, and secretion, as well as biliary excretion, based on the physicochemical properties of the toxicant.^27,2^

Based on data acquired from *in vitro* studies, literature reports, or estimates derived from simulations, the initial model parameters are determined. *In vitro* to *in vivo* extrapolation (IVIVE) is used in PBPK systems to predict the concentration–time profiles of the drugs within the plasma or tissue of an organism without the need for *in vivo* data, and that correlation-based method is beneficial in the field of drug discovery where a large number of drug candidates are required to be analyzed.^4^ After the model is constructed, the verification of the optimized parameters can be carried out by comparing it with an independent data set.

### Nanoparticle-Specific Parameters in the PBPK Modeling of NPs

When constructing PBPK models for the nanoparticles, nanoparticle-specific parameters such as the Hill coefficient, maximum uptake rate in phagocytic cells (PCs), nanoparticle release constant, phagocytic cells (PCs) release constant, partition coefficient, and permeability coefficient between blood and tissue should be included in the model.^2^

Endocytosis of the nanoparticles by phagocytic cells (PCs) is elucidated using both a linear equation and the Hill function, and according to the simulation results in the literature, the Hill function provides more accurate predictions.^17^ The Hill function is described by the following equation:^17^
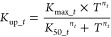
where *K*_up_*t*_ is the uptake rate parameter of NPs by PCs in the organ *t* at time *T*, in 1/h, *K*_max_*t*_ is the maximum uptake rate constant of phagocytic cells, in 1/h, *K*_50_*t*_ is the time to reach the half of *K*_max_*t*_, in h, *T* is the simulation time, in h, *n_t_* is the Hill coefficient (unitless).

To attain simulated *in vivo* data in PBPK modeling of nanoparticles, some of the parameters such as permeability data, transporter-mediated uptake, liver transporter kinetic data, and metabolic enzymes (CYPs) can be obtained by *in vitro* experiments, and the resultant data can be used in different stages of ADME processes of the PBPK model.^2^ Nanoparticle-specific descriptions and exemplary values of nanoparticle-specific parameters used in PBPK modeling including Hill coefficient (*n*), tissue/plasma partition coefficient (*P*), permeability coefficient (χ_a_), maximum uptake rate constant (*K*_max_), release rate constant (*K*_out_), time for reaching half-maximum uptake rate (*K*_50_), and elimination rates for the bile or kidney (Cl_bile_ or Cl_kidney_) in various compartments in body, depending on their availability in the published literature, are shown in Table 1. Further detailed information on the NP related parameters is given under the section Physiologically Based Pharmacokinetic (PBPK) Modeling Applications of Nanoparticles.

**Table 1:** NP-Specific Descriptions and Exemplary Values of Parameters Used in PBPK Modeling

parameter (unit)	description	nanoparticle and size	lung	liver	spleen	kidney	tumor	heart	brain	rest of body	reference
*n* (unitless)	Hill coefficient	mPEG5k-9.09%-80 nm^a^	0.001	0.001	1.62	0.04	0.005	0.07	N/A	0.5	Li et al., 2021^13^
		mPEG5k-28.57%-80 nm^a^	0.001	0.001	1.6	0.04	0.005	0.07	N/A	1	
		mPEG5k-9.09%-200 nm^a^	0.002	0.02	1.6	0.04	0.2	0.07	N/A	0.5	
		mPEG5k-28.57%-200 nm^a^	0.0002	0.5	0.4	0.04	0.2	0.07	N/A	1	
		mPEG2k-28.57%-200 nm^a^	0.0002	0.9	0.01	0.04	0.2	0.07	N/A	1	
		Superparamagnetic iron oxide nanoparticles coated by gold and conjugated with poly(ethylene glycol) (PEG) (SPIO-Au-PEG NPs)- 38.3 nm	2	0.1	0.1	0.1	N/A	N/A	N/A	N/A	Chen et al. 2022^28^
		Water-dispersible cadmium telluride/cadmium sulfide (CdTe/CdS) QDs-4.2 nm	5	7	2	3	N/A	N/A	N/A	5	Liang et al., 2016^29^
		Polyethylene glycol-coated gold nanoparticles (PEG-coated AuNPs)-13 nm	5	5	5	5	N/A	N/A	N/A	N/A	Lin et al., 2016^17^
		Polyethylene glycol-coated gold nanoparticles (PEG-coated AuNPs)-100 nm	0.1	0.1	0.1	0.1	N/A	N/A	N/A	N/A	
*P* (unitless)	Tissue/plasma partition coefficient	mPEG5k-9.09%-80 nm^a^	0.15	0.08	0.15	0.15	0.15	0.15	N/A	0.15	Li et al., 2021^13^
		mPEG5k-28.57%-80 nm^a^	0.15	0.08	0.15	0.15	0.15	0.15	N/A	0.15	
		mPEG5k-9.09%-200 nm^a^	0.15	0.08	0.15	0.15	0.15	0.15	N/A	0.15	
		mPEG5k-28.57%-200 nm^a^	0.15	0.08	0.15	0.15	0.15	0.15	N/A	0.15	
		mPEG2k-28.57%-200 nm^a^	0.15	0.08	0.15	0.15	0.15	0.15	N/A	0.15	
		Cerium oxide (CeO_2_) NPs-25 and 90 nm	0.209	0.209	0.209	0.209	N/A	0.209	0.209	0.209	Li et al., 2016^30^
		Polyethylene on glycol-coated polyacrylamide (PAA–PEG) NPs-35 nm	0.147	0.147	0.147	0.147	N/A	0.147	0.147	0.147	Li et al., 2014^31^
		Stavudine-AuNP (40 nm)	N/A	0.55	0.77	N/A	N/A	N/A	0.46	N/A	Zazo et al., 2022^11^
		Superparamagnetic iron oxide nanoparticles coated by gold and conjugated with poly(ethylene glycol) (PEG) (SPIO-Au-PEG NPs)- 38.3 nm	0.15	0.08	0.15	0.15	N/A	N/A	0.15	0.15	Chen et al. 2022^28^
		Water-dispersible cadmium telluride/cadmium sulfide (CdTe/CdS) QDs-4.2 nm	0.015	0.15	0.15	0.015	N/A	N/A	N/A	0.15	Liang et al., 2016^29^
		Polyethylene glycol-coated gold nanoparticles (PEG-coated AuNPs)-13 nm	0.15	0.08	0.15	0.15	N/A	N/A	0.15	0.15	Lin et al., 2016^17^
		Polyethylene glycol-coated gold nanoparticles (PEG-coated AuNPs)-100 nm	0.15	0.08	0.15	0.15	N/A	N/A	0.15	0.15	
*χ*_a_ (unitless)	Permeability coefficient	mPEG5k-9.09%-80 nm^a^	0.001	0.001	0.03	0.001	0.001	0.001	N/A	0.001	Li et al., 2021^13^
		mPEG5k-28.57%-80 nm^a^	0.001	0.001	0.03	0.001	0.001	0.001	N/A	0.001	
		mPEG5k-9.09%-200 nm^a^	0.001	0.001	0.03	0.001	0.001	0.001	N/A	0.001	
		mPEG5k-28.57%-200 nm^a^	0.001	0.001	0.03	0.001	0.001	0.001	N/A	0.001	
		mPEG2k-28.57%-200 nm^a^	0.001	0.001	0.03	0.001	0.001	0.001	N/A	0.001	
		Cerium oxide (CeO_2_) NPs-25 and 90 nm	0.776	0.776	0.776	0.776	N/A	0.776	6.75 × 10^–7^	0.0171	Li et al., 2016^30^
		Polyethylene on glycol-coated polyacrylamide (PAA-PEG) NPs - 35 nm	1.06 × 10^–3^	1.06 × 10^–3^	1.06 × 10^–3^	1.06 × 10^–3^	N/A	1.06 × 10^–3^	0	8.25 × 10^–5^	Li et al., 2014^31^
		Superparamagnetic iron oxide nanoparticles coated by gold and conjugated with poly(ethylene glycol) (PEG) (SPIO-Au-PEG NPs)- 38.3 nm	10^–3^	10^–3^	10^–3^	10^–3^	N/A	N/A	1.32 × 10^–4^ (MF-)^a^3.51 × 10^–4^ (SMF)^a^	10^–6^	Chen et al. 2022^28^
		Water-dispersible cadmium telluride/cadmium sulfide (CdTe/CdS) QDs-4.2 nm	0.0001	0.001	0.001	0.0001	N/A	N/A	N/A	0.001	Liang et al., 2016^29^
		Polyethylene glycol-coated gold nanoparticles (PEG-coated AuNPs)-13 nm	0.001	0.001	0.03	0.001	N/A	N/A	0.000001	0.000001	Lin et al., 2016^17^
		Polyethylene glycol-coated gold nanoparticles (PEG-coated AuNPs)-100 nm	0.001	0.001	0.001	0.001	N/A	N/A	0.000001	0.000001	
*K*_max_ (h^–1^)	Maximum uptake rate constant	mPEG5k-9.09%-80 nm^a^	1.04	132	65	0.52	15	0.9	N/A	4	Li et al., 2021^13^
		mPEG5k-28.57%-80 nm^a^	0.05	151	83	0.05	15	0.16	N/A	2	
		mPEG5k-9.09%-200 nm^a^	10	560	350	2.2	32	2.57	N/A	51	
		mPEG5k-28.57%-200 nm^a^	1	100	130	0.7	16	0.4	N/A	21	
		mPEG2k-28.57%-200 nm^a^	0.1	280	140	1.5	16	6.94	N/A	28	
		Cerium oxide (CeO_2_) NPs-25 and 90 nm	1.45	1.45	0.518	1.45	N/A	1.45	1.45	1.45	Li et al., 2016^30^
		Polyethylene on glycol-coated polyacrylamide (PAA-PEG) NPs-35 nm	16.1	16.1	0.112	16.1	N/A	16.1	16.1	16.1	Li et al., 2014^31^
		Stavudine-AuNP (40 nm)	N/A	0.15	0.03	N/A	N/A	N/A	7.9 × 10^–3^	N/A	Zazo et al., 2022^11^
		Superparamagnetic iron oxide nanoparticles coated by gold and conjugated with poly(ethylene glycol) (PEG) (SPIO-Au-PEG NPs)- 38.3 nm	0.1	4	10	0.1	N/A	N/A	N/A	N/A	Chen et al. 2022^28^
		water-dispersible cadmium telluride/cadmium sulfide (CdTe/CdS) QDs-4.2 nm	0.0026	0.15	0.09	0.07	N/A	N/A	N/A	0.2	Liang et al., 2016^29^
		Polyethylene glycol-coated gold nanoparticles (PEG-coated AuNPs)-13 nm	0.075	20	40	0.075	N/A	N/A	N/A	N/A	Lin et al., 2016^17^
		Polyethylene glycol-coated gold nanoparticles (PEG-coated AuNPs)-100 nm	0.1	4	10	0.1	N/A	N/A	N/A	N/A	
*K*_out_ (h^–1^)	Release rate constant	mPEG5k-9.09%-80 nm^a^	0.92	5	3	0.085	0.27	0.075	N/A	0.1	Li et al., 2021^13^
		mPEG5k-28.57%-80 nm^a^	4	12	7.2	0.085	1.1	0.075	N/A	0.17	
		mPEG5k-9.09%-200 nm^a^	2	7	3.6	0.085	0.53	0.075	N/A	0.41	
		mPEG5k-28.57%-200 nm^a^	1	9	1.6	0.085	0.53	0.075	N/A	0.94	
		mPEG2k-28.57%-200 nm^a^	1	11	1.4	0.085	0.53	0.4	N/A	0.32	
		Cerium oxide (CeO_2_) NPs-25 and 90 nm	5.30 × 10^–19^	5.30 × 10^–19^	5.30 × 10^–19^	5.30 × 10^–19^	N/A	5.30 × 10^–19^	5.30 × 10^–19^	5.30 × 10^–19^	Li et al., 2016^30^
		Polyethylene on glycol-coated polyacrylamide (PAA-PEG) NPs- 35 nm	4.90 × 10^–19^	4.90 × 10^–19^	4.90 × 10^–19^	4.90 × 10^–19^	N/A	4.90 × 10^–19^	4.90 × 10^–19^	4.90 × 10^–19^	Li et al., 2014^31^
		Superparamagnetic iron oxide nanoparticles coated by gold and conjugated with poly(ethylene glycol) (PEG) (SPIO-Au-PEG NPs)- 38.3 nm	0.005	0.0075	0.003	0.01	N/A	N/A	N/A	N/A	Chen et al. 2022^28^
		Water-dispersible cadmium telluride/cadmium sulfide (CdTe/CdS) QDs-4.2 nm	0.0061	0.011	0.0072	0.002	N/A	N/A	N/A	0.0121	Liang et al., 2016^29^
		Polyethylene glycol-coated gold nanoparticles (PEG-coated AuNPs)-13 nm	0.003	0.001	0.001	0.0004	N/A	N/A	N/A	N/A	Lin et al., 2016^17^
		Polyethylene glycol-coated gold nanoparticles (PEG-coated AuNPs)-100 nm	0.005	0.0075	0.003	0.01	N/A	N/A	N/A	N/A	
*K*_50_ (h)	Time for reaching half-maximum uptake rate	mPEG5k-9.09%-80 nm^a^	0.006	0.001	0.003	0.04	0.02	0.4	N/A	0.5	Li et al., 2021^13^
		mPEG5k-28.57%-80 nm^a^	0.006	0.001	0.003	0.04	0.02	0.4	N/A	1	
		mPEG5k-9.09%-200 nm^a^	0.0052	0.0008	0.003	0.04	0.3	0.4	N/A	0.09	
		mPEG5k-28.57%-200 nm^a^	0.0002	0.46	0.0056	0.012	0.3	0.4	N/A	10	
		mPEG2k-28.57%-200 nm^a^	0.0002	0.04	0.01	0.012	0.3	0.4	N/A	0.4	
		Superparamagnetic iron oxide nanoparticles coated by gold and conjugated with poly(ethylene glycol) (PEG) (SPIO-Au-PEG NPs)- 38.3 nm	24	24	24	24	N/A	N/A	N/A	N/A	Chen et al. 2022^28^
		Water-dispersible cadmium telluride/cadmium sulfide (CdTe/CdS) QDs-4.2 nm	7.5	2.78	1.5	6.82	N/A	N/A	N/A	7.5	Liang et al., 2016^29^
		Polyethylene glycol-coated gold nanoparticles (PEG-coated AuNPs)-13 nm	24	48	48	24	N/A	N/A	N/A	N/A	Lin et al., 2016^17^
		Polyethylene glycol-coated gold nanoparticles (PEG-coated AuNPs)-100 nm	24	24	24	24	N/A	N/A	N/A	N/A	
Cl_bile or_ Cl_kidney_ (L/h)	Elimination rates for the bile or kidney	mPEG5k-9.09%-80 nm^a^	NA	0.0012	NA	0.00012	NA	NA	NA	NA	Li et al., 2021^13^
		mPEG5k-28.57%-80 nm^a^	NA	0.0012	NA	0.00012	NA	NA	NA	NA	
		mPEG5k-9.09%-200 nm^a^	NA	0.0012	NA	0.00012	NA	NA	NA	NA	
		mPEG5k-28.57%-200 nm^a^	NA	0.0012	NA	0.00012	NA	NA	NA	NA	
		mPEG2k-28.57%-200 nm^a^	NA	0.0012	NA	0.00012	NA	NA	NA	NA	
		Superparamagnetic iron oxide nanoparticles coated by gold and conjugated with poly(ethylene glycol) (PEG) (SPIO-Au-PEG NPs)- 38.3 nm	N/A	0.0012	N/A	0.00012	N/A	N/A	N/A	N/A	Chen et al. 2022^28^
		Water-dispersible cadmium telluride/cadmium sulfide (CdTe/CdS) QDs-4.2 nm	N/A	0.000000001	N/A	0.000000001	N/A	N/A	N/A	N/A	Liang et al., 2016^29^
		Polyethylene glycol-coated gold nanoparticles (PEG-coated AuNPs)-13 nm	N/A	0.00003	N/A	0.000003	N/A	N/A	N/A	N/A	Lin et al., 2016^17^
		Polyethylene glycol-coated gold nanoparticles (PEG-coated AuNPs)-100 nm	N/A	0.0012	N/A	0.00012	N/A	N/A	N/A	N/A	

a MF-: No magnetic field; SMF: external static magnetic field; methoxy poly (ethylene glycol)-poly (″ε-caprolactone) (mPEG–PCL).

### Non-Nanoparticle Specific Parameters in the PBPK Modeling of NPs

The input parameters in PBPK models can be divided into different categories: physicochemical properties; drug-biological properties; and anatomic and physiological properties (organ-specific).^2^ Physicochemical properties can be regarded as drug-dependent parameters including the molecular weight, partition coefficient, equilibrium constants, solubility, pH-dependent partition coefficient, and membrane affinity.^2^ Drug-biological properties refer to the properties that are related to both the drug and the organism.^2^ Some examples of these properties are the fraction unbound value of the drug, Michaelis–Menten kinetic parameter constants (*K*_m_ and *V*_max_), and dissociation constant.^2^ The drug-specific parameters are obtained from different *in vitro* studies by extrapolation or by model fitting to the available experimental data.^4^ Organ-specific parameters include organ volumes, blood flow rates, surface areas, tissue composition and expression levels.^2^ For these physiological parameters, a standardized set of values commonly employed in such contexts is utilized. The pharmacokinetic disposition for different populations can be predicted by using some of these above-mentioned properties; for instance expression level information can be predictive information on the determination of gene expression for some of the metabolizing enzymes in different organs for various populations.^2^ The parameters utilized in PBPK modeling along with the application areas are presented in Figure 2.

### Interspecies Scaling Parameters based on Clearance and Human Equivalent Dose

Interspecies scaling techniques have been commonly used in the prediction of pharmacokinetic parameters of different species.^32^ Allometric scaling is an empirical method that is used in interspecies scale-up based on the similarities in the anatomy, physiology and biochemistry of the cross-species in terms of power functions that correlate the physiological parameters with body size. This method has been used in the projection of the human pharmacokinetics for small molecule drugs and also for therapeutic proteins. Allometric scaling has also been widely used in the pharmaceutical industry for the initial decision-making for the various stages in drug discovery and development. The main equation used in the allometric scaling is described as follows:^33^
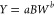
where *Y* is the parameter of interest, BW is the body weight, and *a* and *b* are the coefficient and exponent of the allometric equation, respectively.

Allometric scaling of clearance using a single species to obtain human clearance was carried out in various ways such as based on the body weight of the species or in terms of the physiological factor of the species using different physiological parameters, i.e. liver weight (LW), kidney weight (KW), liver blood flow (LBF), kidney blood flow (KBF), and lymph flow rate (LFR). Some of the allometric scaling to predict human clearance described in the literature studies are shown in Table 2.

**Table 2:** Allometric Scaling to Predict Human Clearance

allometric scaling equation	allometric scaling method	notes	reference
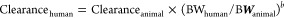	Single species scaling	BW is the body weight, *b* is the allometry exponent. Value of the allometry exponent is 0.60, 0.65, 0.70, 0.75, 0.80, 0.85, or 0.90^33^	(33, 34)
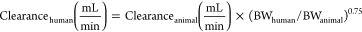	Single species scaling	BW is the body weight	(35)
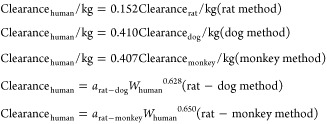	One- or two-species scaling by Tang method	*a*_rat-dog_ is the coefficient for rat-dog method; *a*_rat-monkey_ is the coefficient for rat-monkey method	(36)
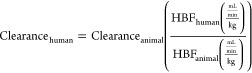	Single species scaling using hepatic blood flow	HBF is the hepatic blood flow	(35)
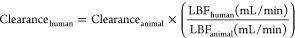	Scaling using liver blood flow	LBF is the liver blood flow, the value of LBF (mL/min/kg) should be multiplied by the corresponding body weight of the species	(33, 36)
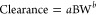	Multiple species scaling by simple allometry (SA)	BW is the body weight, *a* is the coefficient, and *b* is the allometry exponent	(33, 34)
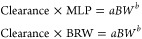	Multiple species scaling by exponent rule-corrected allometry (ROE)	MLP is the maximum life-span potential and BRW is the brain weight of the organism, BW is the body weight, *a* is the coefficient, and *b* is the allometry exponent	(33, 34)
		If *b* < 0.71 in simple allometry, no correction factor is applied; if 0.71 ≤ *b* < 1, MLP is used as a correction factor; if 1 ≤ *b*, BRW is used as an correction factor^33^ If 0.56 < *b* < 0.70, simple allometry is used, 0.71 < *b* < 0.99 or 1.0 ≤ *b* ≤ 1.3 MLP or BRW is used for small molecules^34^	
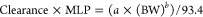	Product of MLP and Clearance (MLP × Clearance)	MLP is the maximum life-span potential and BW is the body weight, *a* is the coefficient, *b* is the allometry exponent, 93.4 is the human MLP in years, and this relation is predicted for the human clearance of Antibody–drug conjugates (ADCs)	(34)
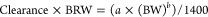	Product of Brain Weight and Clearance (BRW × Clearance)	BRW is the brain weight, BW is the body weight, *a* is the coefficient, *b* is the allometry exponent, 1400 is the human brain weight in grams, and this relation is predicted for the human clearance of Antibody–drug conjugates (ADCs)	(34)
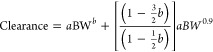	Multiple species scaling by multiexponential allometry (MA)	BW is the body weight, *a* is the coefficient and *b* is the allometry exponent determined from the simple allometry analysis.	(33)
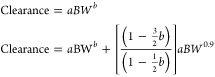	Multiple species scaling by exponent rule-corrected multiexponential allometry (SA+MA)	BW is the body weight, *a* is the coefficient and *b* is the allometry exponent. If *b* < 0.71, simple allometry method is applied with no correction factor; if *b* ≥ 0.71, the multiexponential allometry equation is used.	(33)
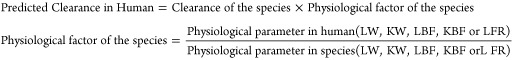	Single species scaling using different physiological parameters	LW is the liver weight, KW is the kidney weight, LBF is the liver blood flow, KBF is the kidney blood flow, and LFR is the lymph flow rate	(37)

Human Equivalent Dose (HED) prediction can be carried out by a variety of methods, as shown in Table 3: linear method based on clearance values, exponential method based on clearance values, the method based on body weight, the method based on predicted human clearance and the method based on correction factor.

**Table 3:** Human Equivalent Dose (HED) Prediction Methods

HED prediction equation	HED prediction method	notes	reference
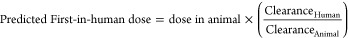	Dose prediction by linear method based on clearance values	Dose and clearance values are in absolute numbers, not normalized to body weight	(37)
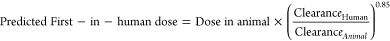	Dose prediction by exponential method based on clearance values	Dose and clearance values are in absolute numbers, not normalized to body weight	(37)
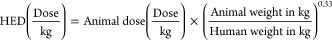	Dose prediction based on body weight	Dose values are normalized to body weight	(37−39)
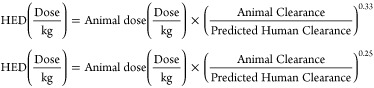	Dose prediction based on predicted human clearance	Dose values are normalized to body weight	(37)
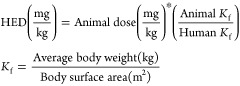	Dose prediction based on correction factor	*K*_f_ is the correction factor; dose values are normalized to body weight	(38−40)

### Benefits and Drawbacks of Allometric Scaling Approach in Predicting Drug and Nanomaterial Clearance

Clearance is regarded as the most important pharmacokinetic parameter that affects the distribution of the drug. The prediction of human clearance is very crucial in early drug discovery and development, but there is a conflict on the most suitable method to attain the clearance data.^35^

Among different pharmacokinetic parameters belonging to a drug or a nanoparticle, systemic clearance is the most scaled parameter from preclinical data using allometry. The implementation of statistical analyses of allometric scaling functions and variables for the pharmacokinetic parameter of nanoparticles has not been possible due to the limited number of nanoparticle-based clinical investigations and also the difficulty of attaining appropriate multispecies pharmacokinetic data from the literature.^41^ In contrast to small molecule drugs, limited studies on the multispecies data of nanoparticles prevent the identification of correlations between nanoparticle-specific properties and biases in allometric relationships.^41^ Furthermore, studies have shown that despite having similar physicochemical characteristics, certain nanoparticles, for example, three types of PEGylated liposomal NPs loaded with anticancer drugs, exhibit different allometric coefficients and exponents. This discrepancy arises due to their varying pharmacokinetic profiles within the same species, even when administered at comparable doses.^42^

Making generalizations based on interspecies allometric relationships of nanoparticles even with similar physicochemical characteristics is not effective due to showing different dispositions and pharmacokinetic profiles.^41^ It should be also stated that the simple allometry approach carried out for drugs and nanoparticles can cause predictive errors when applying interspecies scaling to humans since scaling up the physicochemical, anatomical, physiological, and biochemical properties based on body weight does not always show accurate results.^41^ The possible reasons that result in predictive errors in allometric scaling to humans can be regarded as the differences in interspecies hepatic metabolism, species-dependent pharmacokinetic profiles for nanoparticles or nanoparticles showing a nonlinear relationship with the dosage, species-specific differences in protein interactions with nanoparticles and drugs, and the requirement of at least three or more species of animals to obtain robust correlation.^41^

To improve the predictive performance of the interspecies allometric scaling in terms of clearance of the drug or nanoparticles, some modifications have been carried out to the standard power-law function by the inclusion of different factors such as maximum life-span potential (MLP), organ weights (e.g., brain weight, liver weight, etc.), *in vitro* metabolic data (e.g., from liver hepatocyte or microsome assays), blood flows (e.g., liver blood flow of monkey), and correction for protein binding (differences in fraction unbound values), etc. Each of the allometric scaling methods used for the prediction of the clearance has their benefits and drawbacks in terms of different aspects as the summary table is provided in Table 4.

**Table 4:** Benefits and Drawbacks of Several Allometric Scaling Methods Employed for the Prediction of Clearance (CL)

allometric scaling methods based on clearance	benefits	drawbacks
Simple Allometry (SA)	Frequently used, simple, and fast method^32^	Poor for the drugs that are significantly eliminated by biliary excretion, renally eliminated with significant reabsorption, drugs with extensive metabolism, and combination of hepatic metabolism and renal excretion^32^
	Least successful in predicting clearance for the drugs metabolized in the liver with a low extraction rate^32^	
	Good for the drugs that are renally excreted drugs and low-protein bound drugs^32^	
Product of CL and Maximal Life Potential (CL × MLP) or CL and brain weight (CL × BRW)	Improves the predictive performance of scaling compared to SA^32^	Not suitable for large animals because of higher prediction errors^32^
	Enhances the prediction for the drugs that are hepatic metabolized and renally eliminated^32^	Mathematical manipulation; does not have any physiological relevance^32^
Scaling by rule of exponents (ROE)	Enhances the prediction greatly compared to SA, CL × MLP and CL × BRW^32^	Results in prediction errors in larger preclinical animals (and humans)^42^
	Improves the predictive power for the drugs and nanoparticles with extensive hepatic metabolism^42^	The accuracy of the prediction depends on the type of species used^32^
		Unable to be used for renally excreted drugs^32^
		At least three animal species are needed to correctly apply ROE^34^
Scaling by multiexponential allometry (MA)	Successful in predicting human clearance^46^	When used with preclinical data from more than 3 species accuracy improves^47^
		Prediction accuracy is higher than SA but lower than ROE^48^
Monkey Hepatic Blood Flow Approach	Successful method to predict human clearance compared to SA^47^	Least successful for renally excreted drugs^32^
	Only requires CL of monkey^48^	Not a commonly used method; the prediction is based on an empirical assumption^48^
	Successful in the prediction for the biliary excreted drugs^32^	
Scaling from one or two animal species	Requires only clearance from one or two species^48^	Neglects interspecies differences in metabolism and protein binding^48^
Vertical allometry and fraction unbound (FU) corrected intercept method (FCIM)	Improved accuracy due to taking into account the interspecies differences in protein binding^48^	Requires the data for the fraction unbound value in plasma^48^
	Prediction accuracy is enhanced for the drugs having high extraction rate^32^	
Incorporation with physicochemical properties of a drug	Predicts clearance from molecular weight, clogP, and the number of hydrogen bond acceptors^49^	Requires comprehensive computational effort
	Predicts human clearance using regression methods, multiple linear regression (MLR) analysis, partial least-squares (PLS) method, and artificial neural network (ANN)^49^	
Incorporation of IVIVE by using *in vitro* data (hepatocytes, microsomes, etc.) and interspacing scaling	Improved prediction of *in vivo* CL in different species by adjusting CL for relative metabolism rates obtained *in vitro*(32)	Can only be applied to the drugs that are metabolized in liver; not suitable for the drugs that are renally eliminated or eliminated both by hepatic and renal excretion^32^
	Requires only *in vitro* data;^48^ provides the inclusion of interspecies differences in hepatic metabolism in predicting CL^32^	

Some of the allometric scaling methods are regarded as impractical due to different aspects; for example, although improving the predictive performance of the scaling compared to simple allometry, the product of clearance and maximal life potential (CL × MLP) or clearance and brain weight (CL × BRW) methods are regarded as providing only mathematical adjustments without physiological significance.^32^ Pure *in vitro* to *in vivo* extrapolation (IVIVE) methods may yield inaccurate results when extrapolating drug-specific parameters such as *K*_m_ and *V*_max_, derived from cellular components (e.g., hepatocytes) and subcellular components (e.g., microsomes) of organs. This inaccuracy arises because these methods often neglect the effects of physiological processes and the interactions between the drug and both the intracellular and extracellular environments.^43^ Additionally, when incorporating *in vitro* data from components like hepatocytes and microsomes to obtain *in vivo* clearance values, the predicted clearance can be misleading for drugs that are renally eliminated or eliminated by both hepatic and renal excretion. This method is valid only for drugs that are metabolized only in the liver.^32^

The scaling method of rule of exponents (ROE) proposed by Mahmood and Balian in 1996^44^ was applied in allometric scaling studies based on nanoparticle pharmacokinetics and resulted in successful outcomes for PEGylated liposomal anticancer drugs.^42^ In the study of pharmacokinetics using the allometry on PEGylated liposomal anticancer drugs with the addition of MLP to the standard allometric equation, the linear correlation of the allometric scaling function in preclinical animal models was enhanced.^42^ In a previous pharmacokinetic study, the clearance data of colloidal tumor necrosis factor-alpha (TNF)-gold nanoparticles in rabbit, rat, and oncology patients were scaled by allometric scaling using a power model and were not found predictive for the human clearance.^45^ To improve the predictability of the human clearance, Mahmood’s ROE model with the inclusion of brain weight as a multiplication factor to clearance was applied, and hence the correlation coefficient for the interspecies clearance model was improved significantly.^45^ The ROE method has been reported to significantly improve the predictive power of the allometric relations for drugs and nanoparticles with extensive hepatic metabolism; however, this method resulted in predictive errors in larger preclinical animals and humans contingent on the types of species used in fitting.^41^

### *In Vivo* Conditions of Nanoparticles and Their Challenges on ADME and Scaling

Since the measured physicochemical properties of nanoparticles such as size, surface charge, and surface chemistry are not the only factors in the determination of *in vivo* disposition properties of nanoparticles, the correlation of *in vitro* nanoparticle properties with *in vivo* disposition is a compelling issue.^4^ Also, other than particle properties there are other determinants to account for the *in vivo* conditions of nanoparticles such as aggregation and biocorona formation.^4^ When the nanoparticles are introduced into biological fluids, biomolecular corona formation occurs, where the process consists of the spontaneous adsorption of proteins, lipids, metabolites, nucleic acids, and sugar moieties on the surface of the nanoparticles.^50^ The biomolecular corona formation of the nanoparticle surfaces changes the physiochemical properties and influences their subsequent interactions with the biosystems, and hence the content of the biomolecular corona determines the effects of the nanoparticles on the biological systems.^50^ Most of the studies focus on the adsorption of the proteins regarding the formation of protein-corona on the nanoparticle surfaces.^50^ Protein-corona formation may provide beneficial outcomes for disease diagnosis and personalized nanomedicine when desired adjustments are done for the optimization of cell internalization or for the improvement of *in vivo* biodistribution.^50^ However, by affecting the disposition and pharmacokinetic profiles of nanoparticles, the protein-corona formation may limit the access and distribution of nanomaterials to their targeted sites of action and excretion.^41^

The formation and stabilization of biomolecular coronas within the body are generally directed by the thermodynamics and the biological environment; these corona formation processes are independent of the physiological rates within the organism including the basal metabolic rate (BMR). This situation makes the prediction of pharmacokinetic parameters and simple extrapolations of the nanoparticles debatable.^32^ To attain better nanoparticle characteristics *in vivo*, the allometric scaling approaches (allometric extrapolation of pharmacokinetic parameters) should be improved.^41^

A pharmacokinetic model on the potential fate of NPs *in vivo* took the rate processes dependent on basal metabolic rate and the dynamics of protein corona into account.^51^ The time scales on which metabolic and physiological processes occur and the protein-corona formation kinetics differing from each other that consequently result in mismatches were assumed to contribute significant differences in interspecies extrapolations of nanoparticle biodistribution.^51^ Across two species, the effect of corona formation on nanoparticle biodistribution was highest when the half-life of the corona transition is similar to the geometric average of NP half-lives of the two species.^51^ The interspecies differences in the blood proteomes can bring about different compositions of protein-coronas which may lead to inaccuracies in the extrapolation results.^52^ Consequently, since allometric power-law functions are not adequate to describe the effect of protein-corona formation on the surface of the nanoparticles, novel strategies that combine experimental *in vitro* and *in vivo* data and *in silico* modeling methods by taking into account biocorona dynamics are crucial to extrapolate the pharmacokinetics of nanoparticles.

The pharmacokinetics of tumor necrosis factor-alpha (TNF)-gold nanoparticles that were predicted for rats and rabbits were scaled to humans by an allometric scaling approach.^45^ The allometric correlation between the clearance value and the body weight was found to be moderately predictive in humans.^45^ Following adjustments on the simple allometric equation in the determination of clearance by using the multiplication factor as brain weight, the scaling prediction was significantly improved.^45^ However, since preclinical data from at least three species were required to predict the exponent of powers in model scaling to determine the clearance by regression analysis, better predictions can be obtained when the number of species is increased in the model for TNF-gold nanoparticles. A study carried out a simple allometric scaling approach to deferoxamine-based nanochelator (DFO-NP) in rats to predict pharmacokinetic parameters in mice and humans.^53^ This method predicted the serum concentration–time profile of DFO-NPs to be very close to the experimentally determined values in mice, and nonlinear disposition and absorption models were validated for DFO-NPs for multiple doses across the species.^53^ The pharmacokinetic profile of DFO-NPs in humans was predicted by carrying out allometric scaling by applying clinically meaningful dosages, and pharmacokinetic simulations were carried out demonstrating improved pharmacokinetics by a nanochelator compared to PK profiles after typical infusion protocols of DFO.^53^ However, some limitations have been reported such as the requirement of setting up DFO-NP-specific allometric exponents for all the mechanisms incorporated in the pharmacokinetic model to increase the accuracy of the prediction.^53^ Another constraint in the model was explained as using simple allometry in the scaling of nonlinear pharmacokinetics may be unreliable.^53^

In the allometric scaling of nanomaterial pharmacokinetics, another challenge encountered for many of the intravenously administered nanoparticles is the nonlinear or dose-independent disposition and pharmacokinetic profiles.^41^ There is no theoretical framework to scale nonlinear pharmacokinetic parameters by carrying out allometric approaches because the pharmacokinetic parameters display relationships with various independent variables, such as dosage and body weight, that can be described by multiple linear regression analysis rather than the power-law function.^41^ These nonlinearities generally stem from the saturation of elimination mechanisms associated with the phagocytic cells of the mononuclear phagocyte system (MPS) organs, such as spleen, liver, bone marrow, and lungs, within the framework of the nanoparticles cleared by hepatic elimination after intravenous administration.^41^ A previous study compared the disposition of pegylated liposomal anticancer drugs across species of male and female mice, rats, dogs, and patients with refractory solid tumors, by substituting the physiological parameters of body weight in the allometric equation to predict clearance by the factors related to the mononuclear phagocyte system (MPS).^42^ The parameter that showed the strongest correlation to liposomal clearance was found as the total monocyte count, and this parameter was attributed as a better physiological variable for the allometric scaling of pegylated liposomes in animals and humans.^42^ Hence, it was interpreted that MPS-associated factors such as monocyte count may enhance the clearance prediction in humans.^42^

To sum up, although substantial efforts have been spent on the improvement of nanoparticle interspecies allometric scaling in terms of their pharmacokinetic parameters, there are some restrictions to derive such correlations across species. The protein-corona formation on the surface of the nanoparticles when introduced into biological fluids, the nonlinear disposition and pharmacokinetic profiles with prolonged circulation time of nanoparticles, and their comprehensive interactions with MPS organs especially the liver can be regarded as outstanding challenges for scaling nanomaterial pharmacokinetics. Moreover, different physicochemical properties such as size, shape, surface modification of the nanoparticles and distinct pharmacokinetic profiles make the development of generalized allometric relationships difficult for different types of nanoparticles. Investigating different allometric scaling methods by analyzing multispecies data and determining the advantages and disadvantages of these relationships is crucial. With careful consideration of the requirements of each method, improvements in these correlations play a critical role in developing enhanced scaling approaches for nanoparticle pharmacokinetics.

### *In Vitro* to *In Vivo* Extrapolation (IVIVE) Studies and Challenges

The use of *in vitro* and *in silico* methods in the determination of the pharmacokinetic properties or toxicology levels of the compounds has attracted great attention when the scientific, ethical, and practical issues considered in *in vivo* methods are taken into account. While *in vitro* experiments facilitate fast application across large chemical sets, elucidation of the data obtained from these methods is sometimes challenging because of the mechanistic nature of many assays.^54^ In this aspect, *in vitro* to *in vivo* extrapolation (IVIVE) has become a promising computational tool to provide *in vivo* predictions from results obtained by *in vitro* and *in silico* studies. The term IVIVE was traditionally used in the literature to refer to the estimation of *in vivo* whole-organ ADME properties by scaling from the properties obtained from *in vitro* methods whose results were used in the construction of bottom-up pharmacokinetic (PK) and PBPK models.^55^ The ADME parameters that are commonly determined via *in vitro* analysis methods are plasma protein binding fraction, hepatic metabolism, and intestinal absorption.^55^ Current i*n vitro* methods also facilitate the calculation of the parameters such as, glucuronidation, renal clearance, extrahepatic clearance, and tissue or blood partition coefficients.^55^ Lately, IVIVE methods have been used to describe the conversion of *in vitro* concentration of the molecule related to its bioactivity into an external exposure level.^55^ This procedure, also referred to as reverse dosimetry, includes the determination of the required drug exposure level that results in the plasma or tissue concentration equivalent to the *in vitro* concentration by carrying out PK modeling.^55^ At this point, some important factors should be examined while conducting IVIVE approaches such as the selection of the kinetic model and parametrization of exposure, the modeling technique of the test article, and the selection of *in vitro* assays.^56^ Although IVIVE methods for dosimetry generally consider that the chemicals in *in vitro* systems behave the same way as in the blood or tissue of an organism, the presence of various kinetic factors in *in vivo* studies such as chemical binding to proteins and lipids in the culture medium, degradation processes, internalization processes in the cultured cells, etc. make this assumption inappropriate.^55^ Hence, the *in vitro* bioactivity concentration of the drug needs to be adjusted according to these kinetic factors.

For the characterization of the metabolism, literature data are generally used to obtain metabolic clearance data.^57^ In most of the published data, subcellular fractions such as microsomes, cytosol, or primary cell monoculture systems have been used in *in vitro* determination of the clearance values.^57^ The major challenge encountered when using literature data in the parametrization of IVIVE models is that *in vivo* metabolism processes include integrated systems that contain interaction and competing reactions, and these may not be fully ensured by the simplified systems used in the *in vitro* studies.^57^ For example, when microsomal fractions are used in the determination of metabolic data of a compound via *in vitro* studies, this method is limited only to specific processes such as phase I cytochrome-P450-mediated oxidative reactions, epoxide hydrolases, carboxylesterases, and phase II metabolism via glucuronide conjugation.^57^ However, some of the enzymes such as soluble phase II enzymes including glutathione S-transferases and sulfotransferases are neglected.^57^ In this aspect, comprehensive research has shown that freshly isolated primary hepatocytes, which express most of the proteins in the human liver including the proteins related to the membrane transport, metabolism, and receptor-mediated processes are promising systems both for the determination of hepatic metabolism and metabolite mediated effects of compounds.^57^ However, this system has also drawbacks such as a limited time of viability in suspension form, and a rapid change in enzyme activity in the plated form of primary hepatocytes.^57^ Novel techniques should be developed to overcome these limitations to stabilize the hepatic phenotype for longer durations.

While pharmacokinetic (PK) modeling has been generally used to estimate plasma or tissue concentrations following *in vivo* exposure, recently PK models have been used for reverse dosimetry approaches, which refer to the exposure dosage prediction, that provides specific plasma or tissue concentrations equal to the bioactive concentrations resulting from *in vitro* assays.^56^ In this aspect, several parameters such as metabolic pathways, exposure routes, tissue compartments, systemic clearance, plasma protein binding, and the dosage regimen should be carefully taken into account. Research has shown that one-compartmental models that provide rough estimates with minimal input parameters are not sufficient to differentiate between various exposure routes.^56^ Hence complex multiple-compartment and PBPK models have been shown to describe complex biological processes of *in vivo* systems better, but with requirements of more specific input parameters such as tissue-specific physiological and metabolic parameters and tissue-to-plasma partitioning coefficients.^56^ In a study that has used an (IVIVE)-linked mechanistic PBPK framework for modeling liver transporters and their interaction with liver metabolizing enzymes, area under the plasma concentration–time curve (AUC), maximum concentration (*C*_max_), and the time to reach *C*_max_ (*t*_max_) values were obtained within 2-fold of the observed data between the dose range of 10–80 mg of rosuvastatin, and the validated model was shown to be satisfactory for integration of wide range of *in vitro* and *in vivo* data.^58^

One of the challenges in the applications of IVIVE approaches arises from the *in vitro* toxicity testing. It is very important in *in vitro* toxicity testing to differentiate between the specific disruption of biomolecular targets or pathways and the generalized disruption of cellular machinery which results in cell stress and cytotoxicity.^55^ Investigations on the general cell stress response and cytotoxicity analyses are significant to distinguish cytotoxic bursts of nonspecific mechanisms such as necrosis and regenerative proliferation from the specific effects on the particular molecular targets.^55^ A recent study developed a Bayesian multispecies PBPK model for interspecies extrapolation and IVIVE, and incorporated toxicogenomic dose–response data into the PBPK model to determine chemical risk assessment.^59^ In that study, perfluorooctanesulfonate (PFOS) was used in the determination of risk assessment, and results showed reference doses (RfDs) for the most sensitive pathways and diseases were found similar to the recent European Food Safety Authority’s guidance values.^59^ These results show that for human health risk assessment of the chemicals, the usage of toxicogenomic dose–response data in combination with PBPK modeling is a promising tool, being a nonanimal alternative to traditional risk assessment.^59^

In the case of nanomaterials, the quantification of an *in vivo* toxicokinetic (TK) profile from *in vitro* studies is challenging unless appropriate IVIVE methods are applied due to the dosimetry consideration between *in vitro* and *in vivo* experimental designs.^60^ In *in vitro* studies, the exposed dose of the nanomaterials to the cell medium is not equal to the dose that is delivered to the cell because of the agglomeration, aggregation, diffusion, and sedimentation properties of the nanomaterials.^60^ Moreover, protein-nanoparticle corona formation should also be considered due to the differences in the type of proteins present in the cell culture media *in vitro* and the proteins present in the plasma *in vivo*.^60^ The delivered dose of the nanomaterials to the target organ in *in vivo* testing is affected by the physiochemical properties such as size, zeta potential, surface functionalization, and agglomeration state.^60^ Also, these properties affect the cellular uptake mechanism and degree of internalization of the nanomaterials inside the cell. To sum up, the determination of target organ exposure to the nanomaterials is challenging due to different physicochemical factors dominating *in vitro* cell culture and *in vivo* conditions resulting in differences in nanomaterial properties. Thus, the application of proper IVIVE methods is significant when using *in vitro* dosimetry and toxicity data to determine the *in vivo* toxicity and TK profiles. In this regard, PBPK modeling can be a promising approach in the determination of TK profiles of nanomaterials, and it facilitates the prediction of target organ dosimetry by correlating external doses administered through various routes of administration. Moreover, it allows for extrapolation across species exposure paradigms and from *in vitro* to *in vivo* settings.^60^ A recent study has used nanomaterial IVIVE for cellular uptake and toxicity data and incorporated it into the PBPK model for the toxicity assessment of gold nanoparticles (AuNPs).^61^

While many PBPK modeling approaches like IVIVE, interspecies extrapolation, and route-to-route extrapolation have been carried out for small molecules, their applicability for nanomaterials has been debated due to various factors related to nanomaterials such as nanoparticle–protein corona formation in different biological fluids. Hence, systematic and appropriate methodologies of PBPK modeling should be developed for nanoparticles.

## Physiologically Based Pharmacokinetic (PBPK) Modeling Applications of Nanoparticles

The use of nanoparticle-based drug delivery systems facilitates the development of therapeutics with enhanced solubility, improved therapeutic index, and improved targeting of the diseased cells.^5^ Among the various advantages of encapsulating drugs into nanoparticles are better control of pharmacokinetic properties of therapeutic substances such as circulation half-life and release characteristics, and restricting the drug interactions with the healthy tissues.^62^ Physiologically based pharmacokinetic modeling has risen to prominence as a robust quantitative tool, owing to its capability to precisely simulate the disposition of nanoparticle or nanoparticle-drug hybrid systems within the body. Such precise modeling holds paramount importance in advancing the development of nanoparticle-based drug delivery systems as well as in assessing the safety, efficacy, and quality of nanoparticle-drug systems. In addition to providing a more mechanistic approach to the determination of drug disposition for individual tissues and organs, PBPK modeling and simulation also facilitate the prediction of human pharmacokinetics from preclinical investigations.^11^ PBPK modeling enables the study of the biokinetics of nanoparticle-drug systems to model past experimental data and also to figure out the suitable drug dosage by carrying out simulations.^2^

**Figure 3:**
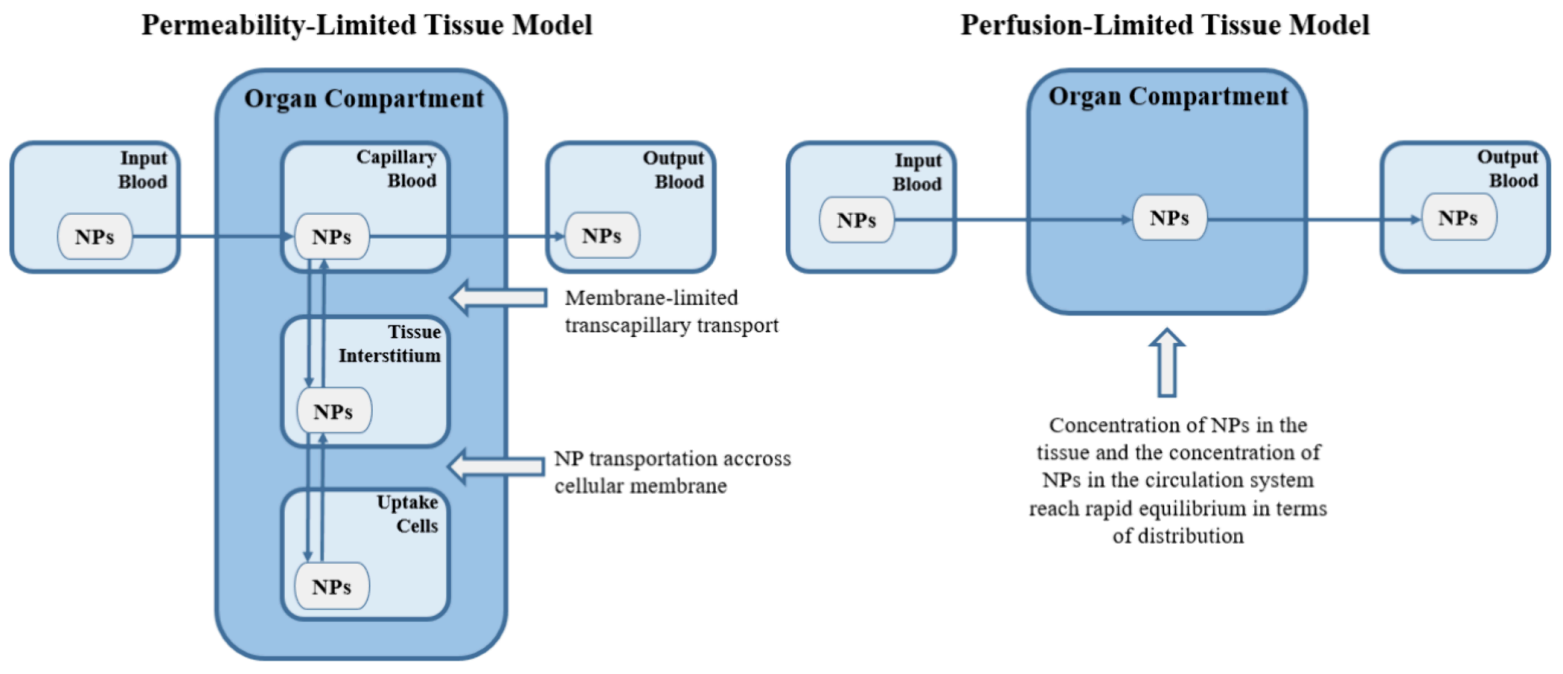
Perfusion-limited and permeability-limited tissue models. Reproduced with permission from ref (62) with minor modifications in the scheme (figure was redrawn and the wordings were paraphrased). Open Access. CC-BY 4.0 license. Copyright: © 2022 by the authors. Licensee MDPI, Basel, Switzerland. URL: https://www.mdpi.com/1422-0067/23/20/12560. No endorsement.

Different from the traditional one-compartment pharmacokinetic modeling, PBPK modeling allows the inclusion of more physiological processes. Each compartment in PBPK models is explained as a permeability-limited model (diffusion-limited or membrane-limited model) or perfusion-limited model (flow-limited model).^3,17,62−64^ In the PBPK model structure of NPs, both permeability-limited (membrane-limited) and perfusion-limited (flow-limited) models can be used to explain the transfer of NPs from the blood to the tissue compartments.^17^ In the permeability-limited model the main barrier of the nanodrug complex is regarded as tissue cell membranes, whereas in the perfusion-limited model, the only limiting factor of nanodrug complex penetration through tissue cell membrane is regarded as blood perfusion. In the perfusion-limited model, it is assumed that the transportation of the nanoparticles into tissues is very fast, and the equilibrium between blood and tissue can be obtained instantly.^62^ In this model, nanoparticle transportation into the tissues depends on its blood supply.^62^ In the permeability-limited model, a membrane is assumed at the capillary or cellular membrane, or both.^62^ In this model, NP transportation across cellular membranes depends on endocytic uptake mechanisms (i.e., phagocytosis, macropinocytosis, and receptor-mediated endocytosis) and exocytic release.^62^ Perfusion-limited and permeability-limited tissue models are represented in Figure 3.

While the exchange of small molecules between tissue and blood is governed by perfusion (flow), the transfer of nanoparticles between blood and tissue is mainly constrained by permeability. Hence, while constructing nanoparticle-based PBPK models, the consideration of permeability (diffusion) limited processes is crucial to obtain accurate results. In the literature, the membrane-limited models were well described for the smaller-sized nanoparticles, e.g. 13 nm of PEG-coated gold NPs (AuNPs),^17^ whereas the perfusion-limited models were more suitable for the larger-sized nanoparticles, such as 100 nm of PEG-coated gold NPs (AuNPs).^17^ The biodistribution of AuNPs was assumed to be controlled by two main processes; the first was the capability of the nanoparticles to cross the capillary membrane of the organs, and the latter was the internalization of the nanoparticles by the process of endocytosis.^17^ The rate of change of the number of NPs in PCs was assumed to be equal to the internalization rate from the tissue (or blood) minus the release rate from the PCs back to the tissue (or blood). The actual uptake rate of the NPs by PCs depended on the uptake rate parameter and the amount of NP for uptake. The equations describing the rate of uptake, release, and mass transfer of small-sized AuNPs in the PCs are shown below:^17^
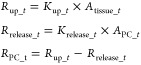
where *R*_up*_*t_ is the uptake rate of the NPs from tissue to PCs in organ *t*, in mg/h, *A*_tissue_*t*_ is the amount of NPs in the tissue subcompartment in organ *t*, in mg, *R*_release_*t*_ is the release rate of the NPs from the PCs to the tissue in organ *t*, in mg/h, *K*_release_*t*_ is the release rate parameter of NPs in the organ *t*, in 1/h, *A*_PC_*t*_ is the is the amount of NPs in the PCs in organ *t*, in mg, *R*_PC_*t*_ is the rate of change in the mass of NPs in the PCs, in mg/h.

In the permeability-limited (membrane-limited) model, each of the compartments contained vascular and tissue spaces and the kinetics of the NPs in the capillary blood, and the tissue of each organ should be described separately. Two kinetic parameters were considered in the modeling of mass transfer between two subcompartments: the ability of AuNPs to diffuse through the capillary wall of the organs and the degree of endocytosis process from the tissue or blood to PCs. The kinetics of the nanoparticles (13 nm AuNPs) in the subcompartments is described as follows:^17^
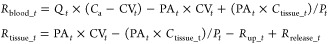
where, *R*_blood_*t*_ is the rate of change of the amount of AuNPs in the capillary blood subcompartment of the organ *t*, in mg/h, *R*_tissue_*t*_ is the rate of change of the amount of AuNPs in the tissue subcompartment of the organ *t*, in mg/h, is the blood flow rate to the organ *t* in L/h, *C*_a_ is the AuNP concentration in the arterial blood of organ *t* in mg/L, CV_*t*_ is the AuNP concentration in the venous blood of organ *t* in mg/L, PA_t_ permeability area cross product between the capillary blood and the tissue of the organ *t*, in L/h, *C*_tissue_*t*_ is the AuNP concentration in the tissue subcompartment, in mg/L, *P*_*t*_ is the tissue to plasma distribution coefficient for the organ *t*, unitless.

**Figure 4:**
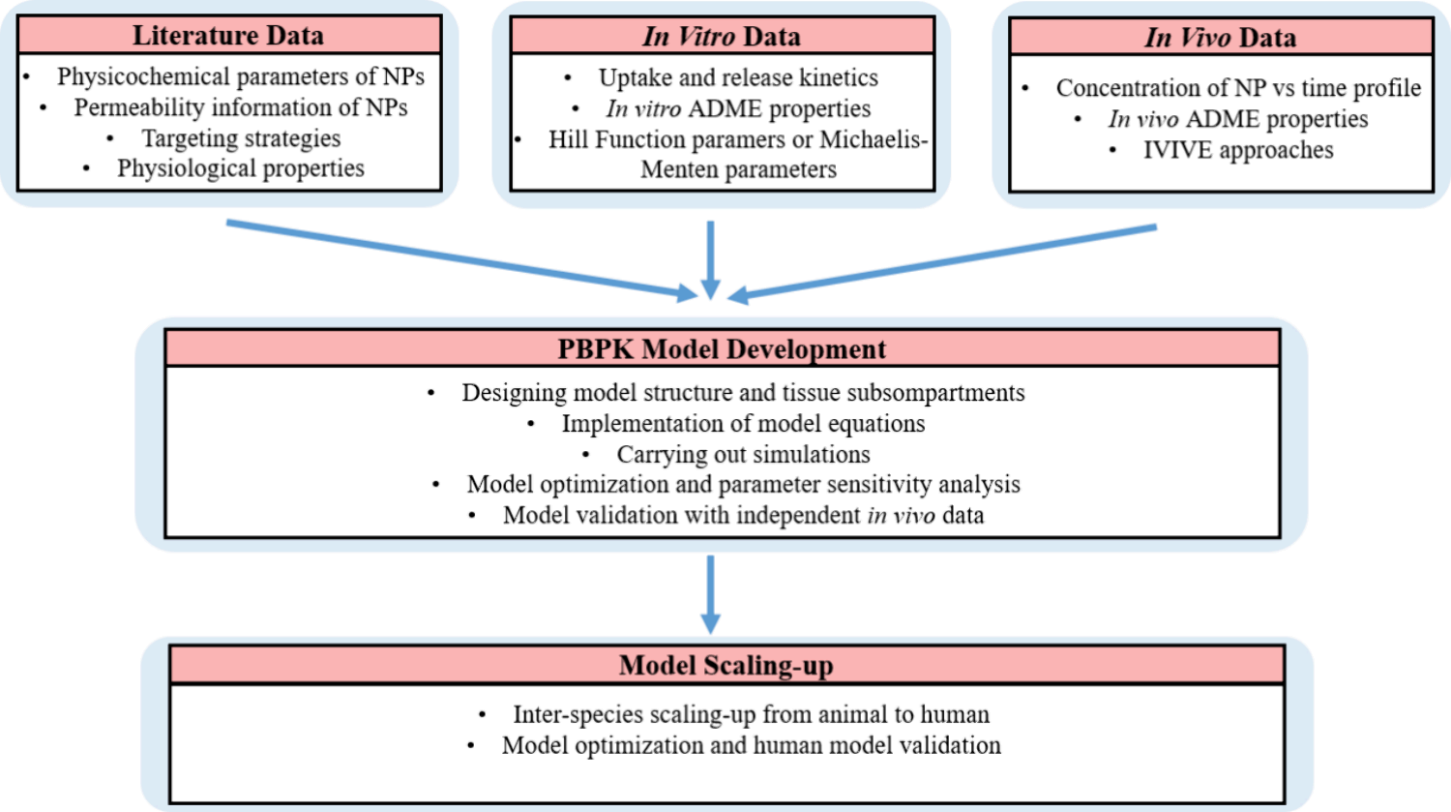
PBPK model development scheme of nanoparticles.

The PBPK model development scheme of nanoparticles is represented in Figure 4. The previously published PBPK models for nanoparticles are summarized in Table 5, with selected notable examples detailed below. In the PBPK model constructed for methoxy poly(ethylene glycol)-poly(″ε-caprolactone) (mPEG-PCL) NPs which were intravenously administered to mice, while using quantification data and direct visualization of specific organs, the PBPK model was focused on the phagocytosis process to reveal nanoparticle kinetics within and among the organs in mice.^13^ The developed model integrated the cellular processes such as phagocytosis and the enhanced permeability and retention (EPR) effects. Both permeability-limited and membrane-limited models were incorporated within the PBPK model, and the results showed that the membrane-limited model was more appropriate to characterize the experimental data. The phagocytic cells (PCs)-PBPK model showed better performance than the enhanced permeability and retention (EPR)-PBPK model, and this model enabled the quantitative representation and prediction of concentration–time profiles and degree of exposure for the mPEG-PCL NPs in blood and different organs.^13^

**Table 5:** Summary of Previously Published PBPK Models for Nanoparticles

type of the nanoparticle	nanoparticle name and physicochemical properties (if available)	modeling species	administration route	data available for model development and validation and software for simulation	PBPK model description	reference
Metal-Oxide NPs	15–150 nm of nano-TiO_2_ NPs	Mice and Rats	Intravenous (IV), oral, and dermal administration	• Organ titanium concentration from toxicokinetic data	• Permeability (membrane) limited model included	Bachler et al., 2015^77^
				• Modified from the literature model^18^	• Two processes are simultaneously considered: NPs to cross the capillary wall of the organs and to transport from blood to tissues by phagocytosis in the mononuclear phagocyte system (MPS).	
				• Model was validated by comparing simulated organ levels with independent *in vivo* experimental data.^75,76^	• The size and crystalline structure of nano-TiO_2_ had a minor effect on the biodistribution; NPs agglomerate at high internal exposure *in vivo* and are internalized by macrophages in the MPS.	
Metal-Oxide NPs	25 and 90 nm Cerium oxide (CeO_2_) NPs	Rats	Inhalation	• Amount of CeO_2_ in lungs, accumulated feces, extrapulmonary organs, gastrointestinal (GI) tract data	• Model was based on PBPK of previously the published version of the study for intravenously injected nanoparticles.^31,78^ Deposition in the respiratory system and transfer to the gastrointestinal (GI) tract was involved for the inhalation exposure.	Li et al., 2016^30^
				•The PBPK model was carried out in Berkeley Madonna version 8.3.18 (Berkeley, CA) and acslX version 3.0.2.1.	• In the model, both permeability and perfusion limited models were used.	
				•The PBPK model successfully predicted biodistribution of CeO_2_ in various organs and identified that most of the nanoparticles that were not eliminated by feces were internalized by the phagocytizing cells (PCs) in the pulmonary region.		
Inorganic NPs	Cerium oxide (CeO_2_) NPs	Rats	Intravenous (IV)	• Various organs (liver, kidneys, brain, spleen, lungs, blood, heart, GIT, rest) concentration of NP data from experimental study was used for calibration and optimization of the model.^79^	• PBPK model was constructed based on the model introduced by the previous literature study.^30^	Kasyanova et al., 2020^69^
				• Model was validated using independent biodistribution study.^80^	• Model included the nanoparticles uptake by phagocytes, as well as different ways of NP removal from the body.	
				• PBPK simulations of CeO_2_ NPs were carried out using Matlab Simbiology R2018b software.	• PBPK model sufficiently described and predicted the biokinetics of intravenously administered CeO_2_ NPs in the body of rats.	
NP-drug or NP-biochemical conjugates	110 nm of Dexamethasone-loaded block copolymer nanoparticles (Dex-NPs)	BALB/c mice	Intravenous (IV)	• Plasma, liver, spleen and kidney concentration data	• Perfusion-limited model applied to tissue compartments and an additional theoretical “other” organ compartment which is connected to the liver, spleen, and kidney included. This compartment stood for the loss of the administered dose injected.	Gilkey et al., 2015^82^
				• Simulations were produced using the experimental data of the previously published *in vivo* study.^81^	• Simulation results were in line with the literature data.^81^	
				• Simulations were conducted via MATLAB.		
NP-drug or NP-biochemical conjugates	25 nm of AuNPs capped with epigallocatechin gallate (EGCG-AuNP) or 19.62 nm of AuNPs capped with curcumin Curc-AuNP	Mice and Rats	Intraperitoneal (IP) Injection	• Amount of NP in heart, kidney, liver, lung, spleen, stomach data	• MPS and whole lymphatic system was included in the model.	Aborig et al., 2019^25^
				• The PBPK model was built and evaluated in MATLAB R2017a using the Intiquan toolbox (IQMTools v1.2.2.2) by Henning Schmidt	• Model evaluation was carried in order to extrapolate biodistribution to other species using experimental data of previous studies.^83,84^	
				• Model parametrization was done using PK-Sim	• In the model, organism-specific parameters were adapted to represent rats, and the rat PBPK model was obtained.	
					• Strong uptake was observed in the liver and spleen in which extravasation and phagocytosis were major mechanisms.	
Organic NPs	Methoxy poly(ethylene glycol)-poly(″ε-caprolactone) (mPEG-PCL) NPs	Mice	Intravenous (IV)	• Blood, heart, liver, spleen, lung, kidney, and tumors concentration of NP data	• Both blood-flow- and membrane-limited models were applied, and the membrane-limited model was found to be the most suitable model to describe their experimental data.	Li et al., 2021^13^
				* The PBPK model of NPs was simulated using Berkeley Madonna v.8.3.23.	• Subcompartment of phagocytic cells (PCs) in the blood compartments was not included since its effect was found to be insignificant in terms of the model fitting.	
					• Enhanced permeability and retention (EPR) effects were integrated into a tumor subcompartment.	
					• Both the PCs-PBPK model and EPR-PBPK model were generated, and the PCs-PBPK model showed better performance.	
Organic NPs	121.8 nm of Poly(alkyl cyanoacrylate) (PACA) loaded with cabazitaxel (PACA-Cbz), and 52.2 nm of LipImage 815	Rats	Intravenous (IV)	• Blood plasma, liver, spleen, lung, brain, heart, and kidney concentration data of cabazitaxel and LipImage 815.	• The model framework was based on a PBPK model developed previously to describe distribution of CeO_2_ nanoparticles.^30^	Minnema et al., 2022^85^
				* Analyses were performed in R using the packages mrgSolve and adaptMCMC.	• Parameter estimation of the PBPK model was carried out by the Bayesian parameter estimation approach.	
					• Results showed that the PBPK model can be adequately parametrized using biodistribution data.	
					• The estimated kinetic parameters may be beneficial to set up a more comprehensive database on kinetic information specific to nanobiomateirals.	
Inorganic NPs	13–20 nm and 80–100 nm of Polyethylene glycol-coated gold nanoparticles (PEG-coated AuNPs)	BALB/c mice	Intravenous (IV)	• Plasma, liver, spleen, kidney, lung concentration data	• Model was calibrated with the literature experimental data.^86^	Lin et al., 2016^17^
				• PBPK model was coded using the acslX	• Transfer of AuNPs from the blood to tissue compartments explained both by perfusion-limited and permeability (membrane) limited models.	
				version 3.0.2.1.	• Both models were successful to simulate 100 nm AuNPs, but the permeability limited model showed better results for 13 nm AuNPs than the perfusion-limited model.	
					• Final model was determined as the permeability limited model to stand for the varied sizes of AuNPs and validated with multiple independent data sets for the NPs of similar sizes.	
Inorganic NPs	1.4–200 nm of AuNPs	Rats	Intravenous (IV), oral gavage, intratracheal instillation, and endotracheal inhalation	• A mechanistic-based PBPK model was calibrated with experimental data sets.	• Tissue distribution of AuNPs was described by taking into account the permeability (membrane) limited transcapillary transport, different distribution of NPs between blood and tissue, endocytosis/phagocytosis using nonlinear functions and exocytic release.	Chou et al., 2022^65^
				• Model was optimized using a Bayesian hierarchical approach with Markov chain Monte Carlo simulations.	• Route-specific approach: a multiroute PBPK model for different sizes of AuNPs	
				• A multiple-linear regression-based quantitative structure–activity relationships (QSAR) model was developed and integrated into the PBPK model	• New route-specific approach had superior performance than the traditional approach.	
				• Model was validated with independent data.	• The size and surface area of AuNPs were the main factors in the determination of endocytic/phagocytic uptake rates irrespective of the administration route.	
				• Web-based nanoparticle interactive physiologically based pharmacokinetic (Nano-iPBPK) interface was developed.	• Zeta potential was a significant parameter to predict the exocytic release rates following IV administration.	
Inorganic NPs	40 nm of AuNPs	Rats	intraperitoneal administration	• Plasma, liver, spleen, thymus, and brain concentration data	• The permeability (membrane) limited model was used in all tissues in the model.	Zazo et al., 2022^11^
				• Simulations were conducted via with Phoenix WinNonlin 64.	• Two tissue compartments: vascular and extravascular which were separated by a cell membrane barrier.	
Inorganic NPs	46, 69, 113, and 162 nm of mesoporous silica NPs	Rats	Intravenous (IV)	• Organ NP concentration data	• Whole-body disposition kinetics was simulated by constructing the PBPK model (without the tumor compartment), and tumor deliverability of NPs was investigated.	Dogra et al., 2020^68^
				• The model was validated with the *in vivo* data from the previous literature study.^87^	• MPS and lymphatic system were included in the model.	
				• The analyses were performed in MATLAB R2018a.	• NP size, degradation rate, tumor blood viscosity, tumor vascular fraction, and tumor vascular porosity are the major factors affecting NP kinetics in the tumor interstitium.	
Inorganic NPs	50 nm of gold NPs (AuNPs) attached with polyethylene glycol (PEG)	Rats	Intravenous (IV)	• Amount of AuNP in blood plasma, liver, spleen, lung, brain, heart, and kidney data	• Physiologically based kinetic (PBK) model framework initially proposed by a previous study was used.^30^	Minnema et al., 2023^88^
				• To parametrize the model, the model parameters were fitted to the *in vivo* biodistribution data.	• The model parameters were compared with PBK parameters for poly(alkyl cyanoacrylate) NPs loaded with cabazitaxel and for LipImage 815 which were studied in a previous work.^85^	
				• Analyses were performed via open-source software R using the program packages mrgSolve and adaptMCMC.		
Inorganic NPs	38.3 nm of Superparamagnetic iron oxide nanoparticles coated by gold and conjugated with poly(ethylene glycol) (PEG) (SPIO-Au-PEG NPs)	Mice	Intraperitoneal (IP) injection	• Blood, liver, lung, and brain concentration of NP data	• Permeability (membrane) limited model was included.	Chen et al. 2022^28^
				• PBPK model was calibrated and validated using the data of *in vivo* experimental results.	• To predict *in vivo* biodistribution of SPIO-Au-PEG NPs, the PBPK model was developed and the dynamic BBB crossing behavior of these NPs was investigated.	
				• Set of ordinary differential equations (ODEs) were numerically solved using COMSOL Multiphysics.	• PBPK model results after incorporating the brain permeability displayed a good agreement with the *in vivo* results	
Inorganic NPs	13 nm of Polyethylene glycol-coated gold NPs (PEG-AuNPs)	Rats	Injection via tail vein	• Blood, liver, spleen, kidney, and lungs concentration of NP data	• The perfusion rate-limited model was used with neglecting active uptake of NPs by phagocytes.	Dubaj et al., 2022^90^
				• The biodistribution curves obtained from simulation were compared with the data in the previous study.^89^	• The membrane permeability coefficient was set to 1 and the absorption rate of nanoparticles by phagocytes was set to 0 for all organs.	
				• The PBPK model was carried out using the SimBiology toolbox (Matlab R2018b).		
Inorganic NPs	168.9 ± 1.1 nm of superparamagnetic iron oxide NPs (SPIONs)	Mice	Intravenous (IV)	• The murine PBPK model validation was carried out by comparing the simulated results with the *in vivo* pharmacokinetic data.	• Novel approach was performed in the integration of *in vitro* experimental data into the PBPK model identifying the uptake of SPIONs in murine macrophage cell line and primary human monocyte-derived macrophages.	Henrique Silva et al., 2017^91^
		Human		• After validation, similar PBPK model for the simulation and distribution SPIONs in human was constructed.	• The modeling of NP penetration into the tissues was carried out by considering passive diffusion of NPs through the capillary endothelium and the active internalization of NPs by the macrophages in the liver, spleen, and lung.	
				* The PBPK model was carried out in Matlab version 2014a using the Simbiology toolbox.	• The murine PBPK model accurately described accumulation of SPIONs in liver, spleen, and lungs.	
					• Human PBPK model showed similar distribution results compared to the murine model with higher NP accumulation in liver and spleen.	
Inorganic NPs	4.2 nm of water-dispersible cadmium telluride/cadmium sulfide (CdTe/CdS) QDs	Mice	Intravenous (IV) and subcutaneous injection	• Blood, liver, spleen and kidney concentration of NP data	• Applicability of the model was evaluated by comparing the PBPK model with the experimental data with 3.5 nm of long-circulating and similar type of QDs.^92^	Liang et al., 2016^29^
				• The PBPK Model of NPs was simulated using Berkeley Madonna version 8.3.18.	• The predictability of the PBPK model for larger QDs (18.5 nm) was evaluated by using the experimental data.^93^	
				• PBPK model was validated using multiple external data sets and under various conditions and in different species.	• The interspecies predictability of the PBPK model was evaluated by comparing the results with the experimental data of 21.2 nm PEG-QDs from rats.^94^	
Inorganic NPs	34 ± 5 nm of Argovit-S Ag NPs	mice	Oral administration	• Brain, lungs, liver, testicles, and blood concentration of NP data.^95^	• A mathematical chamber model describing the biokinetics of Ag NPs in the body of mice was developed based on the previously developed chamber model^96^ and experimental data.^95^	Antsiferova et al., 2023^97^
				• A program in Python was written to solve and visualize the PBPK model of NPs. The integrate.odeint function from the SciPy library was used.	• The obtained numerical parameters were compared with the previous study.^96^	
					• The chamber model described the biokinetics of Ag NPs in a living organism well; numerical differences found with the compared model were attributed to the different dosages of Ag NPs and species differences in model mammals.	

The permeability (membrane) limited approach was considered in the PBPK model developed for 38.3 nm superparamagnetic iron oxide nanoparticles coated by gold and conjugated with poly(ethylene glycol) (PEG) (SPIO-Au-PEG NPs) which were administered to mice via intraperitoneal (IP) injection.^28^ This model predicted *in vivo* biodistribution of SPIO-Au-PEG NPs, and the dynamic blood-brain-barrier (BBB) crossing behavior of these NPs was also incorporated into the model.^28^ After the integration of brain permeability, the PBPK model showed a strong correlation with the *in vivo* data, revealing that the propped model can anticipate *in vivo* biodistribution of SPIO-Au-PEG NPs under an external static magnetic field (SMF).

The traditional route-to-route extrapolation method that is applied for small molecules may not be suitable for the case of nanoparticles. A multiroute (intravenous (IV) injection, oral, intratracheal instillation, and endotracheal inhalation) PBPK model for various sizes (1.4–200 nm) of AuNPs has been constructed for rats.^65^ In the proposed model, the distribution of AuNPs in tissues was elaborated by considering the permeability-limited transcapillary transport, dynamic distribution of NPs between blood and tissue, processes like endocytosis/phagocytosis using nonlinear functions and exocytic release.^65^ Among the two approaches utilized, the novel route-specific approach showed enhanced efficiency compared to the traditional route-to-route extrapolation approach (mainly used for small molecules) implying that the traditional approach was inappropriate for NPs in the development of multiroute PBPK models for NPs.^65^

It has been reported that the physical and chemical characteristics of nanoparticles including size, surface area, and zeta potential have significant impacts on the pharmacokinetics and biodistribution within the body.^65^ Moreover, the size of the nanoparticles is closely linked to their renal clearance or excretion, predominantly because of the limitation on the pore size of glomerular filtration in the kidney.^65^ A particle size-dependent PBPK model for zinc oxide nanoparticles (ZnO NPs) has been developed in mice in order to assess the tissue accumulation characteristics of different sizes of ZnO NPs and zinc nitrate (Zn(NO_3_)_2_).^66^ It was determined that the physicochemical parameters of 10 and 71 nm ZnO NPs and Zn(NO_3_)_2_ in the PBPK modeling played a predictive role in the biodistribution and accumulative levels in living organisms.^66^ The proposed PBPK model in which time-dependent partition coefficients and excretion or elimination rates were used, predicted the ZnO NP and Zn(NO_3_)_2_ distribution in the targeted tissues.^66^ Smaller sized nanoparticles, i.e. 10 nm of ZnO NPs, tended to accumulate in the body for longer periods compared to 71 nm of ZnO NPs and Zn(NO_3_)_2_.^66^ Hence, this study effectively developed a dynamic model that shows promising results in predicting the behavior of slowly decomposed nanoparticles.^66^ A multiroute PBPK model proposed for AuNPs of different sizes ranging from 1.4 to 200 nm showed that the size and the surface area of AuNPs were the primary factors affecting the rates of internalization by endocytosis/phagocytosis irrespective of the administration route.^65^

Nanoparticles that are introduced into the bloodstream are quickly internalized by the phagocytic cells present in organs such as the liver and spleen based on their different physiochemical properties such as size, charge, shape, surface structure, coating, and aggregation status.^67^ Since the most important transportation method for many intravenously administered nanoparticles is regarded as the uptake by the MPS organs such as the liver, spleen, kidneys, lungs, and lymph nodes, more accurate results in pharmacokinetic studies of nanoparticles can be achieved when MPS and the lymphatic system are included in the modeling studies. A recent study developed a mathematical framework to predict whole-body nanoparticle pharmacokinetics and tumor delivery *in vivo*.^68^ The pharmacokinetics of 46, 69, 113, and 162 nm of mesoporous silica NPs was investigated following their intravenous administration to rats. In this model MPS and the lymphatic system were integrated. The size of NPs, the degradation rate, the viscosity of tumor blood, the vascular fraction within the tumor, and the porosity of tumor blood vessels were found to be prevailing factors influencing the NP kinetics within the tumor interstitial space.^68^ Another study investigated the biodistribution of 25 nm AuNPs capped with epigallocatechin gallate (EGCG-AuNP) and 19.62 nm AuNPs capped with curcumin Curc-AuNP via administration of intraperitoneal (IP) injection to mice by carrying out PBPK modeling including MPS and the whole lymphatic system.^25^ Organism-specific parameters in the model were adjusted to reflect those of rats to evaluate the possibility of interspecies extrapolation, and the PBPK model for rats was assessed using the data of citrate-capped AuNPs from the literature.^25^ Successful results were obtained in the prediction of organs by interspecies extrapolation to rats administered by citrate-capped AuNPs.^25^ NPs were strongly internalized by the liver and spleen which indicated the extravasation and phagocytosis were the primary factors contributing to NP uptake.

Another PBPK model was constructed for cerium oxide (CeO_2_) NPs administered intravenously to rats and incorporated the internalization of nanoparticles by phagocytic cells along with the various mechanisms for nanoparticle removal from the body.^69^ The model sufficiently depicted the CeO_2_ biokinetics, and their absorption by phagocytic cells was found a key factor for nanoparticle biokinetics. The results demonstrated that the kinetics of CeO_2_ nanoparticles varied among the tissues in different organs, and CeO_2_ accumulation was primarily observed in liver and spleen. Sensitivity analyses revealed that the partition coefficient between the blood and tissue, the permeability coefficient between capillary blood and tissues, the maximum rate of phagocytic cells, and the maximum capacity for internalization in individual phagocytic cells were the most significant parameters that affect the nanoparticle biokinetics.

One of the limitations in the PBPK modeling of nanoparticles is that the composed model is valid for only that nanoparticle because of the specific physicochemical properties of the nanoparticles and change in parameters such as permeability and macrophage uptake rate according to the type of the nanoparticle.^2^ The investigations in the disposition of the nanoparticle-drug systems have been challenging due to the complexity of their structure and function, and their different characteristics such as size, shape, composition, charge, and surface functionalization which influence the interactions with the biological systems.^4^ This limitation may be overcome by carrying out reparametrization in PBPK modeling of nanoparticles.^2^ As a result, the systematic development of nanoparticle-drug PBPK modeling in a uniting manner is significant and should take the physiological properties, major parameters, and model compartments into account (Figure 4). Since there have been substantial differences in the structure of small molecule drugs and the nanoparticles, some variations arise in the development of PBPK for nanoparticles compared to small molecules. Table 6 presents a brief summary on the differences between PBPK modeling of small molecules and nanoparticles.

**Table 6:** Comparison between the PBPK Modeling of Small Molecules and Nanoparticles

	small molecules	nanoparticles
Modeled molecules	Active ingredient and/or metabolite (s)	Nanoparticle or nanoparticle-drug hybrid system and released drug
Model development	Perfusion (flow)-limited models are more suitable.^3^	Permeability (diffusion)-limited models are more suitable.^3^
Preferred modeling software	Simcyp Simulator, GastroPlus, PKSim/MoBi^62^	MATLAB/SimBiology, Berkeley Madonna, The R Language, acslX, BioUML, PKSim/MoBi^62^
Absorption and uptake by cells	Diffusion and/or active transport by transporters^12^	Paracellular transport,^12^ transcytosis,^12^ M cell uptake,^12^ macrophage uptake and diffusion,^12^ lymphatic uptake,^12^ internalization by phagocytic cells (PCs) by endocytosis/phagocytosis^67^
Biodistribution	Distribute into tissues based on membrane permeability by passive diffusion and active transport via membrane transporters at the blood-to-organ or organ-to-excretion interfaces^98^	Encounter mononuclear phagocyte system (MPS) in the circulatory system that is involved in the biodistribution,^3^ opsonization,^12^ target-mediated disposition,^12^ EPR effect,^12^ lymphatic transport^12^
Metabolism	CYP enzymes and non-CYP enzymes^12^	Extracellular degradation, endocytosis, phagocytosis^12^
	Commonly metabolized in the hepatocytes (parenchymal cells in the liver)^4^	Metabolism primarily occurs in the lysosomes of the MPS^4^
Elimination	Mainly excreted either through hepatic metabolism (liver) or through renal/biliary excretion^99^	Liver and kidney are the organs that are primarily responsible for excretion.^3^
		Slower or very limited renal excretion compared to small molecules due to the larger size.^4^
Protein binding	Binds to plasma proteins after the absorption into systemic circulation	Protein-corona formation on their surface^50^
Extrapolation	Traditional route-to-route extrapolation approaches of PBPK models^65^	Route-specific approach of PBPK models^65^
Allometric scaling	Applicable	Difficult to apply because of nonlinear or dose-independent disposition and pharmacokinetic profiles^41^
Tumor accumulation	Can penetrate normal tissues freely and have no selectivity toward tumor cells^4^	Improved tumor accumulation and tumor selectivity by enhanced permeation and retention (EPR) effect compared to small molecules^4^

### Physiologically Based Toxicokinetic (PBTK) Modeling and Risk Assessment

Toxicokinetics is essential in evaluating the health risks associated with xenobiotics by integrating the principles and approaches in pharmacokinetics and toxicology.^70^ Physiologically Based Toxicokinetic (PBTK) models provide a more comprehensive approach in identifying the chemical concentrations in specific organs or tissues compared to the classical compartmental models.^70^ PBTK models serve as valuable tools for extrapolating data and providing a theoretical foundation for health risk assessment and management.^70^ The internal kinetics of nine chemicals (three endocrine disrupters, three liver steatosis inducers, and three developmental toxicants) were anticipated by using PBTK modeling in data-rich and data-poor conditions, and higher degrees of parametrization complexity were applied.^71^ The results revealed that the parametrization significantly influences the outputs of the model since it can alter the prioritization of chemicals based on internal concentration factors.^71^ A Physiologically Based Toxicokinetic and Toxicodynamic (PBTK-TD) model constructed for the prediction of accumulation and toxicity of Cd and Pb after exposure to zebrafish revealed that the uptake, distribution, and disposition kinetics of Cd and Pb in zebrafish was anticipated accurately.^72^ The toxicodynamic (TD) submodel studies demonstrated that the concentrations of metal in the gill can be a more appropriate indicator of the toxic effect for Cd and Pb than the whole body or other organs. The PBTK-TD model quantitatively predicted the metal toxicity and revealed primary processes by which the uptake and disposition of metals in zebrafish were controlled.

PBPK modeling approaches have been used for the risk assessment of nanoparticles. A PBPK model was developed for ^99m^technetium-labeled carbon nanoparticles (Technegas) following its administration to humans by inhalation.^73^ This PBPK model, which used imaging data to explain the absorption and distribution of nanoparticles, provided a reasonable explanation on the inhalation and distribution of Techngas in humans using Bayesian and MCMC techniques.^73^ To carry out an integrated and probabilistic risk assessment, a PBPK model was developed for AuNPs administered to humans intravenously.^74^ Cell viability assays were used to identify the dose–response relationships in different human cell types. Internal concentrations of AuNPs in the liver, kidney, skin, and venous plasma were quantified by a human PBPK model that was thoroughly validated previously.^74^ Bayesian-based probabilistic risk assessment technique integrated with Monte Carlo simulation was used to depict the probable fractions of human cell death. External exposure levels for AuNPs which induced minimum toxicity were predicted by IVIVE approaches and interspecies extrapolation techniques. The intravenously administered AuNPs at different dosages, determined by allometric scaling or nonscaled values used in rodent studies, possibly exert none to mild nanotoxicity to humans.^74^ Moreover, the importance of adequately deriving human equivalent doses (HED) from doses in rodents was emphasized for future clinical and risk assessment studies.^74^ These types of computational approaches are significant in providing a novel understanding of the safety evaluation and toxicity estimation of nanoparticles.

By integrating physiological aspects of animals with biochemical and physicochemical data, these models offer quantitative insights that are crucial for drug development, toxicology, and personalized medicine. By incorporating the physicochemical characteristics of toxicants, PBPK models provide detailed quantitative predictions of ADME processes. This integration allows for a comprehensive understanding of how toxicants behave in biological systems, supporting risk assessments, regulatory decisions, and the design of safer chemicals and pharmaceuticals.

### Simulation Tools for PBPK Modeling of Nanoparticles

For the description of *in vivo* transport of nanoparticles, there have been several general integration algorithms and programming languages that allow for the coding and solving of the differential equations related to the process.^12^ However, since nanoparticles have complex ADME processes, some of the precoded PBPK modeling tools do not provide adequate flexibility and capability to work with the complex nature of nanoparticles.

The primary used modeling and simulation tools for the PBPK modeling of the nanoparticles are Matlab-Simulink, ACSL/acslXtreme, Berkeley Madonna, and Simbiology MCSim. These software packages have been commonly used for parameter estimation and optimization processes, sensitivity analysis, simulation, and data plotting purposes. Matlab-Simulink, ACSL/acslXtreme, and Berkeley Madonna have integrated specific equation libraries, PBPK modules, and visual graphical interface into the packages. Also, there are other modeling and simulation tools such as ADAPT, Stella, and SAAM II to construct PBPK models of small molecules and therapeutic proteins; however, there is no information available for these tools to be used in the PBPK modeling of nanoparticles.^12^ The built-in algorithms of Simcyp and GastroPlus are not sufficient for NP complexity when working with their free versions.

Comparing the software packages commonly used for the PBPK modeling of nanoparticles in terms of strengths and their limitations, Matlab-Simulink is the most powerful tool and provides graphical representation with Simulink; however, it is expensive and requires more programming skills. ACSL/acslXtreme was also a powerful tool, mainly used in the toxicology area, the upgrading of this tool is no longer provided, and it requires more skills in programming. Berkeley Madonna offers high flexibility to the user, requires fewer skills for programming, and is not expensive and is user-friendly; however, the incorporation of the population variability is hard in this tool. Simbiology MCSim is a free simulation tool that enables one to carry out Bayesian analysis using Markov chain Monte Carlo approach; however, its drawback lies in the absence of a graphical interface.^12^

## Model-Based Methodologies for the Advancement of Disease-Specific Delivery of Nanomaterials

The tumor microenvironment (TME) presents a challenging obstacle to attaining effective nanotherapy-mediated drug delivery to solid tumors.^100^ The drug molecules should overcome various barriers to show therapeutic responses in solid tumors across different physical scales.^100^ TME contains multiple scales such as molecular (nano-) scale (up- and down-regulation of various proteins influencing tumor growth and drug-efflux mechanisms), nano- to microscale (integrating gradients of cell nutrients and oxygen, growth factors and other factors that are crucial for cell-to-cell interaction), microscale (interactions within the acellular stroma section of the tumor), and micro to macro scale (blood supply, lymphatics, organ architecture, and other physiological processes).^100^ Hence the TME becomes heterogeneous regarding the solutes and nutrients (nano- to microscale) as well as the variations in pH levels and cell viability resulting from hypoxia (microscale). Hypoxia has effects on the interactions between cells at the microscale by stimulating the recruitment of immune cells to the tissue and inducing the secretion of cytokines and chemokines at the nanoscale. This heterogeneity in the TME structure strongly influences the therapeutic outcomes. For example, its complexity and three-dimensional structure cause substantial barriers to the systemically administered therapeutics including nanotherapeutics. Also, the cells in the TME including immune cells, macrophages, fibroblasts/myofibroblasts, and endothelial cells actively interface with the tumor cells in most solid tumors and influence cancer cell growth, survival, polarity, and ability to invade surrounding tissues.^100^ The outcomes resulting from the heterogeneity of the TME should be well understood and investigated in the research area of the disease-specific delivery of therapeutics including the nanomaterials, and the development of model-based methodologies is crucial to predict the distribution and site-specific delivery of the substances considering the heterogeneous TME.

The studies on PBPK models of cancer-specific delivery of nanomaterials through heterogeneous tumor microenvironments are very limited. One study investigated the effects of TME heterogeneity on the disposition of nonliposomal doxorubicin (NL-doxo) or polyethylene glycol tagged (PEGylated) liposomal doxorubicin (PLD) nanoparticles and the efficacy on the tumors of the breast.^101^ Genomically validated basal-like *C3(1)-T-Antigen* genetically engineered mouse model (C3-TAg) and claudin-low *T11/TP53*^*Null*^ orthotopic syngeneic murine transplant model (T11), which represent human breast tumor subtypes, were used to analyze whether the heterogeneity of the tumor cells and the TME between breast tumor subtypes have an impact on the tumor delivery and therapeutic effects of NPs.^101^ NL-doxo and PLD were administered intravenously to mice after the tumor size reached a certain level. The area under the curve (AUC) values in plasma for NL-doxo and PLD encapsulated and released doxorubicin were found comparable for the two models, whereas the encapsulated and released doxorubicin AUC value in tumor for PLD was higher in C3-TAg compared to T11. T11 tumors exhibited remarkably higher expression of CC chemokine ligand (CCL) 2 and vascular endothelial growth factor (VEGF)-a, larger vascular quantity and lowered VEGF-c expression compared to C3-TAg. When the two models were compared, PLD was found more efficient than NL-doxo. The results revealed that TME and tumor cell properties in breast cancer influence the efficiency and the tumor delivery of nanoparticles.^101^

Attaining uniform delivery of sufficient quantities of drugs and nanoparticles to the tumors is challenging due to barriers such as antigen expression, the heterogeneous nature of the tumor environment, and its permeability.^102^ The distribution of the nanoparticles in solid tumors has been analyzed by developing computational approaches that investigate drug delivery restrictions stemming from the perivascular areas limiting access to the majority of distant tumor cells. A spatial model of tumor distribution was constructed to simulate the geometric arrangement of tumor vessels and the surrounding tumor cells, and the model was incorporated with a systemic pharmacokinetics model for nanoparticles.^103^ Doxil which is a nanodrug with *liposome* formulation that encapsulates the chemotherapeutic drug, *doxorubicin* was used as a model drug. The effects of various factors including systemic clearance of nanoparticles, tumor blood perfusion, vascular permeability of nanoparticles, diffusion coefficient and nanoparticle release constant in the tumor extracellular matrix, tumor delivery efficacy (ID%), enhanced permeability and retention (EPR) and the magnitude of heterogeneous distribution (H index) were investigated.^103^ The spatial distribution of nanoparticles and their free-form counterparts in the tumors were compared, where high degrees of distributional heterogeneity were obtained for both nanoparticles and free-drug counterparts.^103^ The diffusion coefficient of nanoparticles was determined as the most important factor in decreasing the nanoparticle H index; however, this factor was not prominent on the *H* index of free payloads; the most effective factor in decreasing the *H* index for free payloads was the payload diffusion coefficient.^103^ The factors that increased the free payload distribution were strongly related to the higher drug effectiveness, but the factors that enhanced nanoparticle spatial distributions did not always lead to increased antitumor effects of the administered drug.^103^

In the drug delivery applications of nanoparticles for tumor therapy, the penetration process is challenging due to the complex tumor microenvironments.^104^ A recent study aimed to design nanoparticles and to investigate their transvascular transport in two abnormal tumor microenvironments through a 2-D simulation for drug delivery purposes.^104^ The Computational Fluid Dynamics (CFD) approach was applied to simulate tumor vascular-interstitial models in pancreatic and hepatic tumors, and the influence of the nanoparticles on the velocity profile and pressure gradient in TME was observed.^104^ The findings demonstrated that the nanoparticle velocity in the blood vessels of hepatic tumors was higher compared to the pancreatic tumors because of the smaller diameter of blood vessels in pancreatic tumors.^104^ This result indicated faster delivery of the nanoparticles to the hepatic tumors than to the pancreatic tumors.^104^ Nevertheless, as benign tumors translate into malignant tumors, the diameter of the blood vessel increases, which shows faster delivery of nanoparticle-based drugs to malignant tumors compared to benign tumors.^104^ This study assessed the effect of different TME on the transport of therapeutic agents by carrying out simulations for both pancreatic and hepatic tumors, which have different fluid flow patterns.^104^

## Pharmacokinetic Studies for Specialized Cases

The development of PBPK modeling to predict the pharmacokinetic properties of various drug-like compounds is highly significant for investigating and anticipating the safety of these drugs/nanoparticles under specific individual conditions, such as pregnancy and lactation, as well as assessing their safety for fetuses. Besides the determination of drug safety during drug development, the extrapolation of the administration dosage from nonpregnant subjects is also important considering the physiological changes that take place during pregnancy. The modifications of the body composition in different compartments/organs during the pregnancy influence the digestive, cardiovascular, and renal systems within the body, and this results in the altered pharmacokinetic properties of the API (active pharmaceutical ingredients).^105^ The excretion of the API from the body usually takes place through renal mechanisms after metabolic processes or as an unchanged drug.^105^ Glomerular filtration rate (GFR), fraction unbound (plasma free fraction), and kidney volume are some of the altered physiological parameters that affect the renal excretion of the drug during pregnancy.^106,107^ The variations in the blood flow rates, enzyme expression levels, size of tissues, plasma protein binding, glomerular filtration rates, and other parameters that are affected during the pregnancy were considered by carrying out investigations for both fetal and maternal PBPK models.^105^ A PBPK model was developed to predict the maternal and fetal pharmacokinetics during different stages of the pregnancy, and the model was validated by using the clinical data for the antibiotics excreted via the renal route and commonly used during the pregnancy, which are cefuroxime (CFX) and cefazolin (CZ).^105^

The nanomaterials can cross the placenta and reach the fetus.^3,108−110^ The exposure to nanoparticles in pregnancy may cause damage and fetotoxicity.^111^ The primary factor that leads to the nanoparticle-induced cytotoxicity is the direct translocation of nanoparticles from maternal circulation across the placental barrier into the fetus that is growing.^112^ The stage of embryonic or placental maturation and the nanoparticle surface functionalizations (ferritin, PEG, and citrate) affected the fetal exposure to 13 nm gold nanoparticles during murine pregnancy.^111^ Surface functionality also influences the transfer and accumulation of the nanoparticles in fetus.^111^ The size of the nanoparticles also affects the maternal-fetal transfer and fetal developmental toxicity.^113^ For instance, ZnO nanoparticles with small diameters (13 nm) were able to cross the intestinal and placental barrier and reached the fetus following oral administration to mice.^113^ 13 nm ZnO nanoparticles caused fetal developmental toxicity; however, 57 nm ZnO nanoparticles and bulk nanoparticles did not cross these barriers and show toxic effects.^113^ Similarly, silver nanoparticles (AgNPs) with a size range of 4–20 nm were rapidly absorbed in the gastrointestinal tract following their oral administration to pregnant rats.^114^ The cumulative excretion values of AgNPs showed a renal excretion level of 8.25%, which was higher than the fecal excretion level of 4.77% after 24 h.^114^ The results showed that AgNPs accumulated within the body of pregnant rats and also transferred to the fetus which may lead to adverse effects.^114^ Despite numerous studies that have been conducted to investigate and predict the effects of nanoparticles on pregnant subjects, there appears to be no NP-PBPK model that has been developed for such specific cases.

## Recent Advances in PBPK Modeling: Artificial Intelligence (AI)-Assisted PBPK Modeling

The prediction of input parameters such as absorption, distribution, metabolism, and excretion (ADME) parameters of new compounds can sometimes be challenging in PBPK model development. This situation raises the requirement of computational models to estimate the input parameter values; hence, integrating PBPK modeling with machine learning (ML) or artificial intelligence (AI)-based computational methods has been considered promising for this purpose. In a recent study, this approach consisted of three stages; the first one is obtaining pharmacokinetic (PK) data consisting of a concentration versus time profile and/or ADME parameters from various databases, the second step is to build ML/AI-based methods to estimate ADME parameters, and the last one is to incorporate ML/AI approaches into PBPK models for the prediction of results of PK analysis such as area under the curve (AUC) and maximum plasma concentration (*C*_max_), etc.^115^

There are several challenges that can be encountered in the application of ML/AI-based methods in PBPK modeling. One of these can be regarded as the limited number of data available in the literature, in which more data points help extend the training set by providing the existence of structural diversity of the compounds in the model, and the prediction accuracy of the model can thus be increased.^115^ Another limitation is that the black box nature of many ML approaches in the decision-making process makes the interpretability of the model inadequate.^115^ Also, although neural-ODE is a promising tool to generate time-series of PK profiles for the new compounds that have limited ADME knowledge, its application requires further research.^115^

One of the challenges encountered in the field of nanomedicine has been considered as the inefficient delivery of the nanoparticles to the particular target in the body where tumors are present. There are a number of PBPK models that have been developed for various types of nanoparticles that are loaded with or without cancer drugs.^116^ However, these studies do not use PBPK modeling methods to analyze the NP disposition in tumor-bearing animals using hundreds of disparate data sets to comprehensively investigate the major factors of NP delivery efficiency to tumors.^116^

A previous literature study identified 200 pharmacokinetic studies (376 data sets in total) for PBPK modeling and simulations for tumor-bearing mice intravenously administered with gold nanoparticles (AuNPs) and various inorganic and organic nanoparticles.^116^ The PBPK model was used to estimate the maximum delivery efficiency of the nanoparticles, and the delivery efficiency at 24 and 168 h post-IV administration of the nanoparticles for different time points, different tumor types, and different types of nanoparticles.^116^ However, the limitation of this model was that it was difficult to experimentally measure some critical kinetic parameters of NPs such as Hill coefficient, maximum uptake rate constant, release rate constant, and time reaching 50% maximum uptake rate in the model, and these parameters were usually obtained by fitting to *in vivo* experimental data.^117^ Hence, this method was also dependent on animal studies, and the PBPK model simulation still corresponded to one specific type of NP based on each training data set, so the model could not be efficiently extrapolated from one nanoparticle to the other type of nanoparticles.^117^ Another PBPK model was developed generating data from a recently published Nano-Tumor Database containing 376 data sets, and using multiple machine learning and artificial intelligence methods the prediction of the delivery efficiency of nanoparticles in tumor-bearing mice was performed.^118^ According to this study, the deep neural network sufficiently predicted the delivery efficiency of various nanoparticles to different tumor types, and the type of cancer was an important parameter in the deep neural network model to estimate the tumor delivery efficiency.^118^ Among the different physicochemical properties of nanoparticles, the zeta potential and the type of core material were found to be the most effective ones in the process of delivery efficiency.^118^

Recent developments in computational methods, machine learning (ML), and artificial intelligence (AI) approaches offer novel tools to resolve the issues in targeted drug delivery applications. Machine learning methods have been implemented to predict some rat and human pharmacokinetic parameters such as *C*_max_ and AUC and concentration versus time pharmacokinetic profiles by using their chemical structure and administration dosage^119,120^ and were also used to estimate the ADME properties for small molecule drugs.^115,121^ A previous study has focused on the AI-assisted PBPK modeling via integration of an AI-based quantitative structure–activity relationship (QSAR) model with the PBPK model with the aim of simulation of the tumor-targeted delivery efficiency and biodistribution of different nanoparticles.^117^ The model results correlated well (*R*^2^ ≥ 0.70 for 133 out of 288 data sets) with the experimentally determined pharmacokinetic profiles of various nanoparticles in tumors following intravenous injection.^117^ The AI-assisted PBPK model provided an efficient screening tool to predict tumor delivery efficiency of nanoparticles based on the physicochemical properties of NPs without depending on an animal training data set.^117^ These types of quantitative approaches that integrate machine learning and artificial intelligence methods with PBPK modeling serve as promising studies to predict the tumor delivery efficiency and tumor disposition profile in the field of cancer nanomedicine.

## Concluding Remarks and Future Aspects

Nanoparticles have been found in extensive applications across various domains, particularly in nanomedicine. Accurately determining tissue and blood dosimetry, as well as concentration profiles of nanoparticles, holds paramount importance in evaluating their safety, efficacy, and potential toxicity. There has been an emerging need for the development of novel methods to evaluate nanoparticle safety and efficacy. Moreover, interspecies and *in vitro* to *in vivo* extrapolations of toxicology and pharmacokinetics data play a pivotal role in the effective development of laboratory studies for humans and appropriate risk assessment of nanoparticles. Thus, this review article aims to consolidate the current state-of-the-art and delineate the research challenges in the field of PBPK modeling of nanoparticles. Recent advancements in computational techniques, along with the emergence of machine learning (ML) and artificial intelligence (AI) methodologies, provide innovative solutions for addressing challenges in targeted drug delivery applications. ML techniques have been utilized to forecast certain pharmacokinetic parameters in rats and humans, as well as to generate concentration versus time pharmacokinetic profiles based on chemical structure and dosage information. Some of these PBPK models are validated and show superior concurrence with the predicted and observed data. However, with further understanding of the pharmacokinetics and the biological effects of nanoparticles on humans and other species, more exact PBPK models can be developed.
